# Proteomic Profile of Reversible Protein Oxidation Using PROP, Purification of Reversibly Oxidized Proteins

**DOI:** 10.1371/journal.pone.0032527

**Published:** 2012-02-28

**Authors:** Ken G. Victor, Joshua M. Rady, Janet V. Cross, Dennis J. Templeton

**Affiliations:** Department of Pathology, University of Virginia School of Medicine, Charlottesville, Virginia, United States of America; Shantou University Medical College, China

## Abstract

Signal transduction pathways that are modulated by thiol oxidation events are beginning to be uncovered, but these discoveries are limited by the availability of relatively few analytical methods to examine protein oxidation compared to other signaling events such as protein phosphorylation. We report here the coupling of PROP, a method to purify reversibly oxidized proteins, with the proteomic identification of the purified mixture using mass spectrometry. A gene ontology (GO), KEGG enrichment and Wikipathways analysis of the identified proteins indicated a significant enrichment in proteins associated with both translation and mRNA splicing. This methodology also enabled the identification of some of the specific cysteine residue targets within identified proteins that are reversibly oxidized by hydrogen peroxide treatment of intact cells. From these identifications, we determined a potential consensus sequence motif associated with oxidized cysteine residues. Furthermore, because we identified proteins and specific sites of oxidation from both abundant proteins and from far less abundant signaling proteins (e.g. hepatoma derived growth factor, prostaglandin E synthase 3), the results suggest that the PROP procedure was efficient. Thus, this PROP-proteomics methodology offers a sensitive means to identify biologically relevant redox signaling events that occur within intact cells.

## Introduction

Propagation of intracellular signals depends largely on post-translational modification of signaling proteins. Many studies have focused on signal transduction mediated by protein phosphorylation. Reversible oxidation of protein thiols on cysteine residues potentially affords a mechanism of signal transduction similar to phosphorylation: addition of bulky, charged moieties (e.g. glutathione) or conformational changes (i.e. intracellular disulfides) can easily be imagined to influence enzyme activities or alter protein-protein or protein-nucleic acid interactions. Indeed, some targets are well characterized. The *E. coli oxy*R transcription factor displays oxidant-induced DNA binding [1]. Our group and others have identified several protein kinases that are both positively and negatively impacted by direct or indirect cysteine thiol oxidation [2], [3], [4], [5], [6]. A biotin-labeling strategy has been applied to analysis of tyrosine phosphatases, known targets of oxidative control [7]. Certainly, other targets of cysteine thiol oxidation remain to be identified, and will help delineate pathways of redox signaling.

The field of thiol redox signal transduction lags behind that of protein kinase signaling largely because the reagents and methods for studying cysteine thiol oxidation are limited. While radiophosphate labeling was central to identifying early targets of phosphorylation, the chemical nature of oxidation events makes a radiolabel approach difficult. Other detection approaches such as anti-glutathione antibodies or fluorescent maleimide probes are beginning to make inroads for redox proteomics. An affinity method to identify cysteine thiol oxidation that manifests as sulfenic acid has been described [8]. Considering proteomic approaches, cysteine residue labeling strategies for mass spectroscopic analysis of protein oxidation have also been described. Several have used stable mass labeling reagents such as the thiol reactive ICAT reagent, and have been applied to model proteins or proteins oxidized *in vitro* [9], [10]. A similar strategy has been applied to proteins from *in vivo* models, for example to mice with over-expressed thioredoxin [11] or germinating barley seeds [12]. To our knowledge, these approaches have all relied upon the extraction of proteins in neutral buffer compatible with ICAT, a maleimide compound that requires neutral pH.

Unfortunately, post-lytic oxidation or rearrangements of oxidized thiols is common because the free thiols on solubilized proteins can be oxidized by air and because thiol oxidations can be passed to acceptor targets after cell lysis. To combat this, the approach of rapidly protonating the free thiol groups of proteins through precipitation with trichloroacetic acid has become essential in the thiol oxidation field [13], [14]. This *in situ* acid precipitation step makes conventional thiol blockade with expensive isotopic labeling reagents impractical. Secondly, we have noted that mass spectroscopic sequencing of peptides bearing large cysteine residue adducts such as biotin maleimide is less efficient than sequencing peptides modified with iodoacetamide (IAA). In the end, we were unable to identify an existing proteomics discovery method that (1) was compatible with TCA quenching of post-lysis oxidation, (2) provided efficient thiol blockade and recovery of target proteins, and (3) enabled efficient mass spectroscopic sequencing.

As a consequence, we have recently developed and described a new approach to identify and purify targets of thiol oxidation that we used to dissect oxidative control of the p38 MAP kinase [15]. Referred to as the Purification of Reversibly Oxidized Proteins (PROP), it is a block-and-switch strategy that is similar to one that was recently described to interrogate the oxidative state of multiple specific cysteine residues from proteins of relatively low abundance [16]. This OxMRM protocol, an elegant isotopic labeling approach that couples the use of deuterated *N*-ethylmaleimide (NEM) with Multiple Reaction Monitoring (MRM) mass spectrometry, provides robust quantitation of oxidation for specific cysteine thiols that are known *a priori*, but it is not suitable for discovering novel sites of oxidation. Our PROP protocol, however, provides a complementary proteomics approach that is designed to uncover unknown sites of cysteine thiol oxidation. Furthermore, the PROP procedure is an improvement on previous proteomic approaches because of an optimized thiol blocking protocol and, more importantly, a novel strategy that purifies the targets of oxidation in a manner that results in unmodified proteins suitable for both immunodetection and mass spectroscopic sequencing.

In this report, we outline the PROP-proteomics method that is suitable for a mass spectroscopic analysis of complex mixtures of proteins with reversibly oxidized thiols. We have applied this procedure to proteins oxidized in living human cancer cells. From this demonstration, we highlight some of the oxidized proteins that are recovered using PROP and, in some cases, identify specific cysteine residues that become oxidized. These results illustrate that coupling the PROP procedure with shotgun or targeted proteomic methods provides a powerful new method for dissecting redox signaling pathways.

## Methods

### Cell culture and sample preparation

1×10^6^ HeLa (American Type Culture Collection, ATCC.org) cells were seeded in wells of two 6 well plates in 2 mL of Dulbecco's Modified Eagle's Medium supplemented with 10% calf serum and penicillin/streptomycin. The cells were incubated overnight at 37°C in a 5% CO_2_ environment. The following day the cells had reached approximately 60% confluence. The wells of one plate were treated with 10 mM H_2_O_2_ in 1 mL of conditioned media for 30 minutes. The control plate was manipulated in parallel with water instead of hydrogen peroxide.

### The PROP procedure

Cultured cells were rinsed twice with HEPES-buffered saline and quenched with 1 mL 10% TCA for 10 minutes to prevent post-lytic oxidation. The fixed cells were rinsed three times with methanol containing 10 mM N-ethylmaleimide (NEM) and were then air-dried. Dry monolayers were dissolved in 250 µL of MLB/G (25 mM MOPS pH 7.1, 5 mM EDTA, 150 mM NaCl, 0.1% Igepal detergent, and 6 M guanidine HCL) containing 20 mM NEM. The lysate was scraped from each well and was transferred into tubes. The lysates were sonicated with three 50% duty cycle pulses with a microtip to shear DNA. Lysates were blocked in MLB/G with NEM for 4 hours. To reduce the oxidized thiols, DTT was added to 50 mM and the lysates were incubated at 50°C for 30 minutes. 50 µL was removed from each sample as an input control. (Input samples were removed for consistency with the standard procedure but were not further analyzed.) The proteins were then precipitated overnight at −20°C by the addition of 4 volumes of methanol. To remove excess reducing agents, the protein precipitates were washed 5 times for 5 minutes each by rotational agitation in 100% methanol and then pelleted by centrifugation at 3220×g for 5 minutes in a swinging bucket centrifuge. The washed and air dried pellet for each sample was re-dissolved in 600 µL of MLB/G buffer (without NEM) containing 10 mg of activated thiopropyl sepharose 6B beads (GE Amersham, Piscataway, NJ) that had previously been washed twice with deionized water, per manufacturer's instructions, to swell and remove additives. The pellet was dislodged in this solution and allowed to dissolve and react overnight with rotational agitation at RT, during which proteins with newly revealed thiols formed disulfide bonds with the thiols on the beads. The beads were then washed twice in MLB/G and three times in MLB to remove all proteins that were not bound to the thiol beads.

To exchange the sample buffer for each of the 12 samples, a series of three washes was then conducted with 100 mM ammonium bicarbonate with respect to the MLB/G buffer. The proteins bound to the thiol beads were eluted by the addition of 25 mM DTT in 200 µL of 100 mM ammonium bicarbonate and incubation at 50°C for 60 minutes. The liberated proteins were separated from the beads and, with the addition of iodoacetamide at a 2.5 molar excess with respect to the DTT, carboxyamidomethylated at RT for 60 minutes in the dark. After quenching this reaction with the addition of DTT, the reduced proteins from each of the 12 samples were digested with 1 µg of recombinant trypsin (Roche, Indianapolis, IN) at RT overnight. The 12 trypsinized samples were then taken to dryness on a SpeedVac and put back into solution with 50 µL. Each sample was then cleaned using a C18 ZipTip pipette tip (Millipore, Billerica, MA) by loading the sample, washing with a 95∶5 solution of (Buffer A):(Buffer B) and then eluting with Buffer B, where the two buffers are as described in the following Mass spectrometry section. The eluted peptides were again taken to dryness and then brought back into solution with 20 µL of Buffer A to prepare for mass spectral analysis.

### Mass spectroscopy

Each trypsinized sample was pressure-loaded onto a self-prepared 100 µm i.d. fused-silica column (Polymicro Technologies, Phoenix, AZ) packed with irregular (5–15 µm, 120 Å) reverse-phase phenyl resin (YMC, Kyoto, Japan) and then connected to a 75 µm i.d. PicoFrit® fused-silica column (New Objective, Woburn, MA) that had a pre-fritted 10 µm tip and had been self-packed with regular (5 µm, 120 Å) reverse-phase phenyl resin (YMC, Kyoto, Japan). Nano-flow electrospray ionization was performed in the positive ion mode with a 2.0 kV spray voltage applied to peptides that were eluted with a flow rate of about 200 nL/min from an HPLC gradient of 0–60% Solvent B in 105 minutes, where Solvent A was 0.2 M acetic acid and Solvent B was 80% acetonitrile in 0.2 M acetic acid.

Briefly, the Thermo LTQ-XL ion trap mass spectrometer (Thermo, San Jose, CA) was operated in the data-dependent mode with an Agilent 1100 HPLC system split to nano-flow. The acquisition duty cycle consisted of an initial MS^1^ centroid scan with a mass range of 350–1800 m/z for all experiments. The 5 most abundant ions were sequentially selected for a Zoom MS^1^ scan acquired in profile with a width of 10 m/z centered on the precursor ion. Each Zoom MS^1^ scan was followed by a MS^2^ CID spectrum of that same precursor. After repeating for each of the top five precursor ions, the cycle repeated. The duty cycle for this data acquisition cycle of 11 mass spectral scans was about 3 s. Further details are provided in Lyons, *et al* [17].

### Data analysis

Data sets were analyzed using a Perl script, dubbed MAZIE, written in our lab that accurately determines peptide charge and monoisotopic mass for each MS^2^ scan precursor ion by analyzing the preceding Zoom MS^1^ scan [18] and then generates a concatenated DTA file used for searching with the OMSSA engine [19]. MAZIE is distributed under the Creative Commons License, and is available, together with its dependencies, at http://faculty.virginia.edu/templeton. Using a decoy strategy, the MS^2^ data was searched as a tryptic digest against a composite database containing the human refseq database, (ftp.ncbi.nih.gov/refseq), and the reversed protein sequences generated by an in-house Perl script. Search parameters were optimized as described previously [17], [18], with the mass of both the precursor and fragment ions treated as monoisotopic with an m/z tolerance of 0.3 Da and 0.5 Da, respectively. A false discovery rate (FDR) was calculated by tabulating the OMSSA search results that identified natural (forward) protein sequences, representing potential real sequence matches, together with those that identified reversed protein sequences that are, by definition, false matches [20]. While we typically use a more stringent FDR as the cutoff for confident identifications, for this work we used a more liberal 3% FDR cutoff in order to include a greater number of scans for spectral counting procedures.

This analysis was automated using a Visual Basic script that amalgamates results from each search into a single spreadsheet, calculates a running FDR, and summarizes peptide and protein identifications that fall within the specified FDR cutoff. The complete results table resulting from this script is included as Table S1; Script S1 contains the Visual Basic script, including instructions for use, and is hereby distributed under the Creative Commons license.

## Results

### The PROP procedure

**Figure 1:**
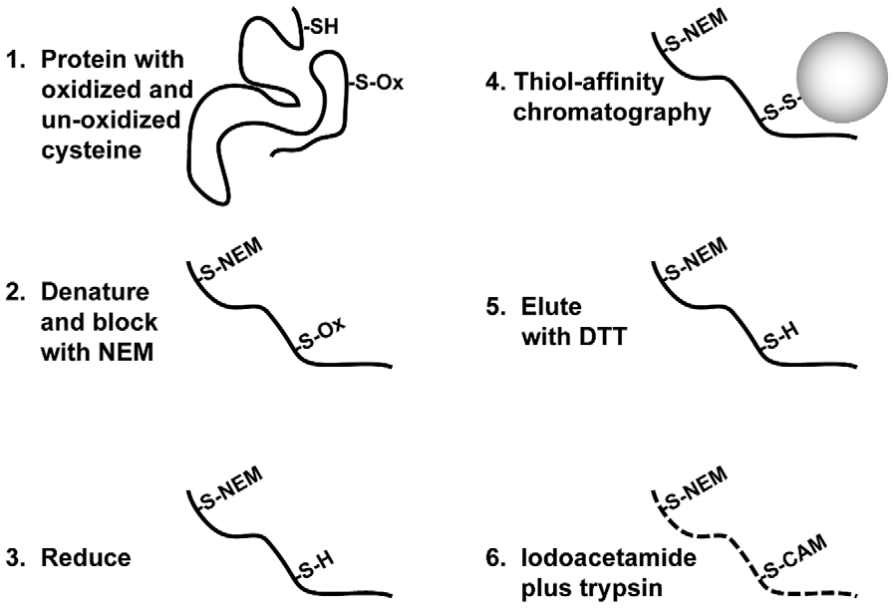
PROP-proteomics procedure. Schematic diagram showing steps involved in the PROP-proteomics procedure that are described in detail in both Methods and Results.

PROP relies on several steps that are diagrammed in Figure 1. In the first step, cells are fixed *in situ* in strong acid, usually 10% TCA, to terminate cellular metabolism and prevent artifactual oxidation post lysis. Second, the acid is washed from fixed cells using methanol containing N-ethyl maleimide (NEM) to begin the irreversible thiol blocking process of the non-oxidized cysteine thiols. The cellular proteins are then dissolved in 6 M guanidine HCl containing additional NEM to complete the covalent thiol blockade. Importantly, the oxidized thiols on proteins are not modified with NEM. The use of denaturing buffer is important because thiols in non-denatured proteins may be inefficiently blocked. In pilot experiments we tested guanidine compared to other denaturing agents, such as urea and SDS. Guanidine was found to be the most efficient denaturant to enable NEM blocking of unoxidized cysteine residues, thereby reducing background in the purification (not shown). Previous work comparing the detergent extracts from both oxidized and unoxidized cells demonstrated that, through this procedure, all of the non-oxidized cysteine thiols were blocked with NEM [15].

Following this exhaustive, irreversible NEM blockade of the non-oxidized cysteine thiols, the excess NEM is inactivated and protein thiol reduction effected by treatment with an excess of dithiothreitol (DTT) at elevated temperatures. Shown as the third step in Figure 1, DTT efficiently reverses S-thiolation (e.g. glutathionylation) and disulfide bonds, and also reverses sulfenic acid (R-SOH), as well as S-nitrosylation. These modifications are collectively referred to as ‘reversible oxidation’. Modification of the PROP procedure using other reducing agents is of course possible, as further considered in Discussion.

Following treatment with DTT, the protein mixture contains guanidine denatured proteins with cysteine residues that had free thiols in the intact cell, now capped with NEM, and free thiols that had been oxidized in the intact cell. Proteins with the newly revealed free thiols, originating from the biologically oxidized sites, are recovered through precipitation with a commercial preparation of activated thiopropyl sepharose beads. Represented as step four in Figure 1, these beads contain a reactive 2-pyridyl disulfide group that is released upon the reaction with the peptide thiols of the protein mixture, resulting in a (Bead)–S–S–Peptide disulfide linkage. Previous studies using oxidized p38 conclusively demonstrated that the thiopropyl sepharose beads efficiently and specifically bind to the DTT-reduced form of the protein [15].

Displayed as step five in Figure 1, the purified, formerly oxidized proteins are then eluted with a DTT-containing sample buffer after the irrelevant proteins are washed away. For the subsequent mass spectral proteomic analysis, the recovered proteins are carboxyamidomethylated with iodoacetamide (IAA) to label the cysteine residues responsible for the thiol bead precipitation distinctly from the NEM blocked cysteine residues. Thus, shown as step six in Figure 1, cysteine thiols derivatized with carboxamidomethyl (CAM) can be identified as those originally biologically oxidized while the cysteine thiols that were reduced at the time of lysis will be labeled with NEM. For this initial study, we then trypsinized the proteins and applied mass spectral shotgun proteomics to the samples without further fractionation because the complexity of the sample has already been far reduced. However, we recognize the value of multidimensional analyses for a more exhaustive analysis.

### Identification of proteins oxidized by H_2_O_2_ in HeLa cells

We prepared samples using PROP from HeLa cells grown in 10% calf serum that were treated with 10 mM H_2_O_2_ or, as controls, cells treated similarly without H_2_O_2_. Both sample sets included six biological replicates to provide a measure of reproducibility, resulting in a total of twelve analyses that are presented individually in the tables of this manuscript and in Table S1. Spectral counting was employed to provide a semi-quantitative measure of relative prevalence of oxidized protein between control and H_2_O_2_ treated HeLa cells [21]. Though spectral counting provides a relatively wide dynamic range and excellent reproducibility with respect to other quantification methodologies [22], caution must be used when interpreting results for proteins with low spectral counts. Because of its inherent limitations, it is difficult to provide a statistical interpretation of relative *abundance* for proteins with spectral counts below four [22], [23], [24], [25].

However, because all of the scans that were tabulated passed a 3% FDR filter cutoff as described in Methods, the confidence in any protein identified with more than one unique peptide is high. Furthermore, a “pValue” parameter was calculated for each of the identified proteins to provide a relative measure of the overall confidence that the protein was in the sample (i.e. a true positive), with 0.00E+000 representing the highest confidence. The protein pValue represents the product of the best (i.e. smallest) eValue score observed for each *unique* peptide sequence for the given protein. Thus, in particular for those proteins identified with only a couple of scans, the protein pValue provides a non-biased means of creating a confidence filter at the protein identification level.

Using a 3% FDR cutoff on the matched scans from the OMSSA searches across the twelve samples, 8089 matched scans identified 697 unique peptides from 286 proteins (see Table S1). After grouping these results by the twelve samples and then clustering the sample results by whether or not they received the H_2_O_2_ oxidation treatment, we identified three patterns of PROP protein recovery. First, about a 30% of the proteins were found at similar expression levels in both sample sets; these likely represent proteins with either internal disulfides or some other reversibly oxidized cysteine thiol(s) that is constitutively present under the conditions of our experiments.

Second, about 50% of the proteins, notably thioredoxin, prostaglandin E synthase 3 and several variants of elongation factor proteins, demonstrated significant increases in spectral counts in the H_2_O_2_ treated sample with respect to the control PROP sample. This category represents proteins that gained reversible cysteine thiol oxidation following the hydrogen peroxide treatment. In many cases, no spectra were identified from the control samples while numerous spectra from two or more peptides were identified from the oxidized samples. Table 1 contains a select list of these proteins that were identified from the PROP protocol, with both the number of unique peptides and the total number of scans identified for each protein indicated for each of the six biological replicates of the two sample sets.

**Table 1:** Selected proteins identified using the PROP analysis on six replicate cell cultures treated or not with H_2_O_2_ that were preferentially identified *after* H_2_O_2_ oxidation treatment of cells.

Protein			H_2_O_2_ †						Untreated†					
gi	Description	pValue‡	1	2	3	4	5	6	1	2	3	4	5	6
50592994	thioredoxin	3.89E−046	4 (35)	5 (46)	5 (30)	5 (31)	4 (23)	4 (23)	1 (2)	2 (4)	2 (5)	1 (1)	1 (2)	1 (1)
4503483	elongation factor 2	1.42E−102	6 (9)	9 (13)	4 (7)	7 (13)	8 (14)	5 (9)	1 (1)	1 (1)	1 (1)	X	X	1 (1)
4503481	elongation factor 1-gamma	9.40E−016	2 (2)	1 (1)	1 (1)	1 (1)	2 (2)	X	X	X	X	X	X	X
15082258	chromobox protein homolog 3	0.00E+000	2 (2)	3 (5)	1 (1)	2 (3)	1 (1)	2 (3)	X	X	X	X	X	X
4503545	eukaryotic translation initiation factor 5A-1 iso B	0.00E+000	8 (28)	8 (23)	12 (26)	10 (21)	8 (27)	10 (24)	X	X	X	X	X	X
4758516	hepatoma-derived growth factor isoform a	0.00E+000	8 (15)	9 (16)	7 (17)	6 (12)	6 (14)	8 (11)	X	X	X	X	X	X
10835063	nucleophosmin isoform 1	0.00E+000	10 (43)	9 (43)	9 (40)	10 (31)	9 (35)	9 (36)	X	X	X	X	X	X
23308579	prostaglandin E synthase 3	9.68E−023	2 (2)	2 (2)	2 (3)	1 (2)	X	1 (1)	X	X	X	X	X	X
5031635	cofilin-1	1.75E−134	9 (16)	7 (11)	6 (9)	8 (12)	8 (16)	6 (13)	X	X	X	X	X	X
10863927	peptidyl-prolyl cis-trans isomerase A	3.39E−087	6 (18)	8 (23)	8 (16)	7 (21)	6 (20)	8 (27)	X	1 (1)	X	X	X	X
4504425	high mobility group protein B1	4.40E−058	4 (5)	5 (8)	4 (5)	3 (6)	3 (4)	2 (3)	X	X	X	X	X	X
4506901	serine/arginine-rich splicing factor 3	9.06E−047	3 (4)	5 (6)	4 (6)	4 (7)	4 (8)	4 (5)	X	X	X	X	X	X
17986258	myosin light polypeptide 6 isoform 1	9.95E−039	3 (14)	4 (13)	3 (9)	3 (13)	3 (12)	3 (10)	X	X	X	X	X	X
4505303	myosin light chain 6B	2.40E−016	2 (2)	2 (4)	1 (1)	2 (4)	2 (4)	2 (4)	X	X	X	X	X	X
4557777	myosin light chain 3	1.40E−013	1 (1)	1 (2)	X	1 (1)	1 (1)	1 (2)	X	X	X	X	X	X
222352151	poly(rC)-binding protein 1	1.98E−036	1 (1)	2 (2)	1 (1)	1 (1)	4 (5)	1 (1)	X	X	X	X	X	X
23308577	D-3-phosphoglycerate dehydrogenase	2.82E−025	X	2 (2)	2 (3)	1 (1)	2 (2)	1 (1)	X	X	X	X	X	X
4505409	nucleoside diphosphate kinase B isoform a	1.62E−016	1 (1)	1 (2)	2 (2)	2 (3)	1 (1)	1 (2)	X	X	X	X	X	X
4504981	galectin-1	5.82E−019	3 (7)	4 (7)	3 (7)	2 (5)	3 (4)	3 (8)	X	X	X	X	X	X
50053795	eukaryotic translation initiation factor 4B	2.84E−018	1 (1)	1 (2)	1 (3)	1 (2)	1 (2)	1 (1)	X	X	X	X	X	X
154355000	far upstream element-binding protein 2	4.85E−014	3 (4)	2 (3)	2 (3)	1 (2)	3 (3)	2 (3)	X	X	X	X	X	X
72534660	serine/arginine-rich splicing factor 7 isoform 1	5.18E−011	1 (1)	2 (2)	1 (1)	2 (2)	2 (2)	X	X	X	X	X	X	X
14277700	40S ribosomal protein S12	1.29E−010	1 (2)	1 (1)	1 (2)	1 (1)	X	1 (1)	X	X	X	X	X	X
4757714	phosphotyrosine protein phosphatase isoform c	5.75E−010	1 (1)	1 (3)	X	1 (1)	1 (1)	1 (2)	X	X	X	X	X	X
4759098	transformer-2 protein homolog beta	2.51E−008	1 (2)	2 (5)	1 (1)	2 (4)	X	X	X	X	X	X	X	X

† Leading number indicates the number of unique peptides for a particular protein that were observed while the number in parentheses indicates the total number of scans that were observed for that protein; in other words, a protein indicated as 3(13) had three unique peptides identified a total of 13 times. All of the scans tabulated passed a 3% FDR filter cutoff. An “X” indicates that no scans were observed that passed the FDR filter. The replicate analysis of 6 independent biological samples for both the control and H_2_O_2_ treated cells are shown. Data referred to in the text combine the replicate analyses.

‡ The pValue represents the product of the best (i.e. smallest) eValue score observed for each *unique* peptide sequence for the given protein. This provides a relative measure of the overall confidence that the protein was in the sample (i.e. a true positive), with 0.00E+000 representing the highest confidence.

We also identified a third category representing about 20% of the proteins that was unanticipated and are presented in a similar manner in Table 2. These were proteins that showed a significant decrease in spectral counts in the H_2_O_2_ treated sample with respect to the control PROP sample. One notable representative of this class of proteins is peroxiredoxin 6, for which a total of 14 spectra were identified from three unique peptides from the control samples while one of those same peptides was seen only once from the H_2_O_2_ treated samples. Both peroxiredoxin 1 and 4 also exhibited a similar expression. Though this category was unexpected, we hypothesize that these represent proteins that are commonly oxidized with disulfide or sulfenic acid in normal cells but that are efficiently modified with higher-order, irreversible oxidized cysteine thiols in cells exposed to H_2_O_2_. This will be discussed further below.

**Table 2 pone-0032527-t002:** Selected proteins identified using the PROP analysis that were preferentially identified *before* H_2_O_2_ oxidation treatment of cells.

Protein			H_2_O_2_ †						Untreated†					
gi	Description	pValue‡	1	2	3	4	5	6	1	2	3	4	5	6
48255889	glucosidase 2 subunit beta isoform 1	5.55E−119	9 (17)	9 (18)	7 (12)	8 (18)	8 (19)	8 (19)	13 (29)	12 (27)	13 (29)	11 (25)	13 (29)	11 (23)
42794771	thioredoxin domain-containing protein 5 iso1	1.73E−049	2 (3)	3 (3)	1 (1)	2 (2)	1 (1)	1 (1)	5 (7)	7 (8)	6 (7)	6 (8)	6 (8)	5 (7)
10835143	complement decay-accelerating factor isofo1	6.62E−045	1 (1)	2 (3)	1 (1)	1 (1)	2 (2)	1 (1)	6 (11)	6 (8)	6 (9)	4 (9)	3 (5)	4 (7)
4505591	peroxiredoxin-1	2.81E−018	1 (1)	1 (1)	X	1 (1)	1 (1)	X	1 (1)	2 (2)	2 (2)	1 (1)	1 (2)	2 (2)
5453549	peroxiredoxin-4	1.20E−007	2 (2)	X	1 (1)	1 (1)	X	X	1 (2)	2 (3)	2 (2)	2 (3)	1 (1)	2 (2)
4758638	peroxiredoxin-6	0.00E+000	X	X	X	X	1 (1)	X	2 (2)	3 (4)	2 (2)	2 (2)	2 (3)	1 (1)
10835165	CD59 glycoprotein preproprotein	1.45E−018	1 (1)	1 (1)	X	1 (1)	X	X	3 (3)	X	1 (1)	2 (2)	X	X
10716563	calnexin precursor	0.00E+000	X	X	X	X	1 (1)	X	3 (4)	2 (2)	2 (3)	2 (2)	3 (3)	2 (2)
38202257	neutral alpha-glucosidase AB isoform 2	0.00E+000	X	X	X	X	X	1 (1)	4 (5)	3 (3)	1 (1)	2 (2)	2 (2)	1 (1)
304376266	putative serine protease 56	0.00E+000	X	X	X	X	X	X	4 (5)	4 (4)	5 (7)	5 (9)	3 (3)	7 (8)
38372919	basigin isoform 1 precursor	3.47E−036	1 (1)	X	X	X	X	X	3 (4)	3 (4)	2 (3)	2 (3)	2 (2)	3 (4)
4758412	polypeptide N-acetylgalactosaminyltransferase 2	1.16E−021	X	X	X	X	X	X	2 (2)	X	1 (1)	1 (1)	2 (2)	2 (3)
40317626	thrombospondin-1 precursor	2.07E−021	X	X	X	X	X	X	X	2 (2)	X	1 (1)	1 (1)	1 (1)
24797067	HLA class I histocompatibility antigen, A-1 alpha	2.82E−007	1 (1)	X	X	X	X	X	1 (1)	1 (1)	1 (2)	1 (2)	1 (1)	1 (2)
17986001	major histocompatibility complex, class I, B precur	1.54E−006	X	X	X	X	X	X	1 (2)	1 (2)	1 (2)	1 (1)	1 (2)	1 (1)
29725609	epidermal growth factor receptor isoform a precur	5.25E−006	X	X	X	X	X	X	1 (1)	1 (2)	1 (1)	1 (1)	X	1 (2)
31542331	protein CYR61 precursor	2.10E−017	X	X	X	X	X	X	1 (1)	1 (2)	1 (1)	1 (2)	1 (1)	2 (3)
116734717	alkaline phosphatase tissue-nonspec isozyme iso1	2.58E−012	X	X	X	X	X	X	1 (1)	1 (1)	X	X	X	1 (1)
4503143	cathepsin D preproprotein	1.04E−024	X	1 (1)	1(1)	X	1 (1)	X	1 (1)	3 (3)	2 (3)	1 (1)	X	X
22538442	cathepsin Z preproprotein	3.78E−014	X	X	X	X	X	X	X	X	X	1 (1)	1 (1)	X
5031863	galectin-3-binding protein	5.66E−004	X	X	X	X	X	X	X	X	X	X	1 (1)	X
17149842	peptidyl-prolyl cis-trans isomerase FKBP2 precur	2.48E−022	X	X	X	X	X	1 (1)	2 (2)	1 (1)	1 (1)	X	1 (1)	1 (1)
167614504	laminin subunit beta-1 precursor	1.29E−016	X	X	X	X	X	X	X	2 (2)	1 (1)	X	X	1 (1)
54607120	lactotransferrin isoform 1 precursor	9.38E−011	X	X	X	X	X	1 (1)	1 (1)	2 (2)	1 (1)	2 (2)	1 (1)	1 (1)

† Leading number indicates the number of unique peptides for a particular protein that were observed while the number in parentheses indicates the total number of scans that were observed for that protein. All of the scans tabulated passed a 3% FDR filter cutoff. An “X” indicates that no scans were observed that passed the FDR filter. The replicate analysis of 6 independent biological samples for both the control and H_2_O_2_ treated cells are shown. Data referred to in the text combine the replicate analyses.

‡ The pValue represents the product of the best (i.e. smallest) eValue score observed for each *unique* peptide sequence for the given protein. This provides a relative measure of the overall confidence that the protein was in the sample (i.e. a true positive), with 0.00E+000 representing the highest confidence.

### Identification of specific cysteine residues as sites of oxidation

By the nature of the PROP protocol, most of the tryptic peptides that are identified will not contain the oxidized cysteine residue(s) responsible for the precipitation of its parent protein. However, from the peptides that are observed, the PROP procedure does present the opportunity to identify the specific cysteine residue that was oxidized in the intact cells following treatment. This is because the protocol uses NEM to irreversibly block non-oxidized cysteine thiols, and iodoacetamide to alkylate and, thereby, distinctly label cysteine thiols derived from formerly oxidized cysteine residues. Identification of these sites of cysteine thiol oxidation by other mass spectroscopic approaches is otherwise quite difficult.

Of the 697 unique peptides identified (using the 3% FDR cutoff) in at least one scan from the twelve runs, 115 (16%), originating from 78 distinct proteins, contained a carboxamidomethyl (CAM) modification on one or more cysteine residues (see Table S1). Table 3 lists a small subset of these peptides that were observed with an oxidized cysteine residue(s) from proteins that were preferentially identified after H_2_O_2_ treatment, many of which are included in Table 1. Table 4 is the corresponding list for such peptides from proteins preferentially identified before H_2_O_2_ treatment. Note that the best representative spectra, with respect to its OMSSA eValue score, from each of the peptides presented in both Tables 3 and 4 would pass a 0.3% FDR cutoff and that many were validated by manual sequencing. In many instances, these represent the first identification of cysteine residues that are potential targets of oxidation in intact cells.

**Table 3 pone-0032527-t003:** Selected peptides that were observed with a cysteine residue that had been biologically oxidized from proteins that were preferentially identified *after* H_2_O_2_ oxidation treatment of cells.

Protein		Peptide		H_2_O_2_ †						Untreated†					
gi	Description	Sequence*	eValue‡	1	2	3	4	5	6	1	2	3	4	5	6
4503545	eukaryotic translation initiation factor 5A-1 iso B	YDcGEEILITVLSAMTEEAAVAIK	4.57E−014	3	0	2	1	2	0	0	0	0	0	0	0
4503545	eukaryotic translation initiation factor 5A-1 iso B	EIEQKYDcGEEILITVLSAMTEEAAVAIK	2.28E−015	0	2	1	1	0	0	0	0	0	0	0	0
4503545	eukaryotic translation initiation factor 5A-1 iso B	KYEDIcPSTHNMDVPNIK	4.35E−018	2	2	2	2	3	2	0	0	0	0	0	0
4503545	eukaryotic translation initiation factor 5A-1 iso B	KYEDIcPSTHNMDVPNIKR	2.40E−012	1	1	1	2	3	2	0	0	0	0	0	0
4504981	galectin-1	FNAHGDANTIVcNSK	3.08E−008	1	1	1	2	0	1	0	0	0	0	0	0
4758516	hepatoma-derived growth factor isoform a	cGDLVFAK	3.72E−003	1	1	1	1	0	1	0	0	0	0	0	0
5031635	cofilin-1	HELQANcYEEVKDR	1.76E−019	0	0	0	0	1	0	0	0	0	0	0	0
5031635	cofilin-1	LTGIKHELQANcYEEVKDR	1.15E−009	2	2	0	2	1	2	0	0	0	0	0	0
10863927	peptidyl-prolyl cis-trans isomerase A	HTGPGILSMANAGPNTNGSQFFIcTAK	6.62E−015	0	0	0	0	0	1	0	0	0	0	0	0
10863927	peptidyl-prolyl cis-trans isomerase A	KITIADcGQLE	1.97E−008	0	0	0	1	0	0	0	0	0	0	0	0
14277700	40S ribosomal protein S12	KVVGcScVVVK	1.29E−010	2	1	2	1	0	1	0	0	0	0	0	0
15082258	chromobox protein homolog 3	LTWHScPEDEAQ	7.03E−009	0	1	1	2	0	2	0	0	0	0	0	0
20149594	heat shock protein HSP 90-beta	LVSSPccIVTSTYGWTANMER	1.18E−012	1	2	1	1	1	0	0	0	0	0	0	0
23308579	prostaglandin E synthase 3	HLNEIDLFHcIDPNDSK	3.13E−014	1	1	2	2	0	1	0	0	0	0	0	0
50592994	thioredoxin	cMPTFQFFK	6.07E−005	2	1	1	2	1	2	0	0	0	0	0	0
50592994	thioredoxin	TAFQEALDAAGDKLVVVDFSATWcGPcK	2.38E−018	15	17	11	5	0	3	0	0	0	0	0	0
148298677	hydroxymethylglutaryl-CoA synthase, cytoplasmic	PGSLPLNAEAcWPK	1.04E−007	0	1	0	1	0	1	0	0	0	0	0	0
148298677	hydroxymethylglutaryl-CoA synthase, cytoplasmic	VTQDATPGSALDKITASLcDLK	3.63E−007	1	0	0	1	0	0	0	0	0	0	0	0
222352151	poly(rC)-binding protein 1	LVVPATQcGSLIGK	7.38E−006	1	1	0	0	2	0	0	0	0	0	0	0

† The total number of scans that were observed for that peptide. All of the scans tabulated passed a 3% FDR filter cutoff. The replicate analysis of 6 independent biological samples for both the control and H_2_O_2_ treated cells are shown. Data referred to in the text combine the replicate analyses.

‡ The eValue represents the best (i.e. smallest) OMSSA eValue score matched to the particular peptide sequence. This provides a relative measure of the overall confidence that the peptide was in the sample (i.e. a true positive), with 0.00E+000 representing the highest confidence.

* Lower case “c” indicates location of carboxyamidomethyl derivatization(s).

**Table 4 pone-0032527-t004:** Selected peptides that were observed with a cysteine residue that had been biologically oxidized from proteins that were preferentially identified *before* H_2_O_2_ oxidation treatment of cells.

Protein		Peptide		H_2_O_2_ †						Untreated†					
gi	Description	Sequence*	eValue‡	1	2	3	4	5	6	1	2	3	4	5	6
10716563	calnexin precursor	cESAPGcGVWQRPVIDNPNYK	3.91E−298	0	0	0	0	0	0	2	1	2	1	1	1
4505591	peroxiredoxin-1	HGEVcPAGWKPGSDTIKPDVQK	1.61E−012	1	1	0	1	0	0	1	1	1	1	2	1
4758638	peroxiredoxin-6	DFTPVcTTELGR	2.58E−008	0	0	0	0	0	0	1	1	1	1	1	1
10835143	complement decay-accelerating factor iso1	cEESFVKIPGEKDSVIcLK	8.81E−011	0	0	1	0	1	0	1	1	1	2	0	1
10835143	complement decay-accelerating factor iso1	EIYcPAPPQIDNGIIQGER	7.05E−011	0	0	0	0	0	0	0	1	1	0	0	0
10835143	complement decay-accelerating factor iso1	LNSASLKQPYITQNYFPVGTVVEYEcRPGYR	2.65E−006	1	0	0	0	0	0	2	2	1	0	0	1
10835143	complement decay-accelerating factor iso1	QPYITQNYFPVGTVVEYEcRPGYR	1.74E−007	0	2	0	1	1	1	2	2	2	2	2	1
10835143	complement decay-accelerating factor iso1	WSTAVEFcK	1.58E−003	0	0	0	0	0	0	1	1	1	0	1	0
304376266	putative serine protease 56	EVLFGVTSWGDGcGEPGKPGVYTR	3.78E−012	0	0	0	0	0	0	0	0	0	0	0	1
304376266	putative serine protease 56	VPLLSTDTcR	5.96E−007	0	0	0	0	0	0	1	1	0	2	1	1
304376266	putative serine protease 56	GSGRPRPQALLQDPPEPGPcGER	1.99E−004	0	0	0	0	0	0	2	0	0	2	1	1
10835165	CD59 glycoprotein preproprotein	FEHcNFNDVTTR	2.75E−009	0	0	0	0	0	0	1	0	0	1	0	0
10835165	CD59 glycoprotein preproprotein	LRENELTYYccK	2.44E−005	0	0	0	0	0	0	1	0	0	0	0	0
31542331	protein CYR61 precursor	GLEcNFGASSTALK	8.87E−009	0	0	0	0	0	0	0	0	0	0	0	1
31542331	protein CYR61 precursor	IcEVRPcGQPVYSSLKK	2.37E−009	0	0	0	0	0	0	1	2	1	2	1	2
38372919	basigin isoform 1 precursor	SSEHINEGETAMLVcK	4.90E−015	0	0	0	0	0	0	1	1	1	1	0	2
17149842	peptidyl-prolyl cis-trans isomerase FKBP2 precur	GWDQGLLGMcEGEK	3.85E−014	0	0	0	0	0	0	1	0	1	0	0	1
29725609	epidermal growth factor receptor isoform a precur	GPDNcIQcAHYIDGPHcVK	5.25E−006	0	0	0	0	0	0	1	2	1	1	0	2
42794771	thioredoxin domain-containing protein5 iso1 precur	VDcTAHSDVcSAQGVR	4.39E−016	0	0	0	0	0	0	0	1	1	0	0	1
42794771	thioredoxin domain-containing protei 5 iso1 precur	IGKVDcTQHYELcSGNQVR	3.09E−004	0	0	0	0	0	0	0	1	1	2	1	1
40317626	thrombospondin-1 precursor	RPPLcYHNGVQYR	6.22E−009	0	0	0	0	0	0	0	0	0	0	0	1
48255889	glucosidase 2 subunit beta isoform 1	cEYLMELMTPAAcPEPPPEAPTEDDHDEL	1.92E−008	0	0	0	0	0	0	1	0	0	0	0	0
48255889	glucosidase 2 subunit beta isoform 1	YEQGTGcWQGPNR	1.26E−009	0	0	0	1	1	0	1	1	2	1	1	1
54607120	lactotransferrin isoform 1 precursor	cGLVPVLAENYK	6.65E−007	0	0	0	0	0	1	1	1	1	1	1	0
167614504	laminin subunit beta-1 precursor	cLYHTEGEHcQFcR	6.38E−005	0	0	0	0	0	0	0	1	1	0	0	1

† The total number of scans that were observed for that peptide. All of the scans tabulated passed a 3% FDR filter cutoff. The replicate analysis of 6 independent biological samples for both the control and H_2_O_2_ treated cells are shown. Data referred to in the text combine the replicate analyses.

‡ The eValue represents the best (i.e. smallest) OMSSA eValue score matched to the particular peptide sequence. This provides a relative measure of the overall confidence that the peptide was in the sample (i.e. a true positive), with 0.00E+000 representing the highest confidence.

* Lower case “c” indicates location of carboxyamidomethyl derivatization(s).

Note that there are also 58 (8%) peptides that contained an N-ethyl maleimide (NEM) modification on one or more cysteine residues (see Table S1). These peptides derive from proteins that contained at least one unoxidized cysteine thiol at the time of harvest, yet were recovered during the PROP process. Thus, they likely represent proteins that have a mixture of both oxidized and unoxidized cysteine residues. Indeed, a number of peptides with multiple cysteine residues were observed with both the CAM and NEM modifications (see Table S1). For example, the peptide TAFQEALDAAGDKLVVVDFSATWcGPcK from the thioredoxin protein was observed a total of 52 times from only the H_2_O_2_ treated samples. Though both of the cysteine residues in this peptide were modified with CAM in 48 of these scans, 3 scans had one of the cysteine residues modified instead with NEM and 1 scan was obtained with both residues modified with NEM. The peptide cMPTFQFFK from the same thioredoxin protein was observed 47 times from only the H_2_O_2_ treated samples also. However, only 9 of these scans were modified with CAM while the remaining 38 scans were modified with NEM. These results suggest that upon H_2_O_2_ treatment, thioredoxin has multiple cysteine residues that are oxidized with variable efficiency.

### Gene Ontology Analysis

After summing the total scan counts across the six biological replicates in the two sample sets, 70 of the 286 proteins that were originally identified had an adjusted 5-fold or greater presence in the H_2_O_2_ treated samples and passed a 1.00E−03 filter on the protein pValue (see Table S1). Note that if a protein was identified with only one scan, this filter on the protein pValue would mean that that scan would have an FDR of less than 0.15% (see Table S1). Using the web-based gene set analysis package WebGestalt [26], a gene ontology (GO), KEGG enrichment and Wikipathways gene enrichment analysis were conducted by submitting the list of the Entrez gene ID of the 70 proteins.

The results from the GO analysis revealed that the proteins that were preferentially found in the oxidized sample set were significantly enriched in both the nuclear mRNA splicing (adjusted p = 3.36e−08) and translation biological processes (adjusted p = 2.98e−05). Table 5 highlights the list of the proteins identified in the H_2_O_2_ treated sample set that were responsible for these results. The most significant result from the KEGG enrichment analysis was the Spliceosome pathway (adjusted p = 2.38e−08) while the Wikipathways analysis determined a significant enrichment in the Translation Factors (adjusted p = 4.58e−11) and mRNA processing (adjusted p = 2.58e−10) gene sets. The full tabular results of all three analyses are included in the Analysis S1.

**Table 5:** Gene ontology (GO) analysis of proteins that were preferentially identified *after* H_2_O_2_ oxidation treatment of cells.

GO Biological Process			Protein				
Translation2.98E−05†	mRNA Processing4.11e−07†	Nuclear mRNA Splicing, via Spliceosome3.36e−08†	Entrez gene	gi	Mass [kDa]	Description	pValue
	X	X	6432	72534660	27.37	serine/arginine-rich splicing factor 7 isoform 1	5.18E−011
	X	X	4869	10835063	32.57	nucleophosmin isoform 1	0.00E+000
	X	X	6434	4759098	33.67	transformer-2 protein homolog beta	2.51E−008
	X	X	3191	52632383	64.13	heterogeneous nuclear ribonucleoprotein L isoform a	1.17E−009
	X	X	6428	4506901	19.33	serine/arginine-rich splicing factor 3	9.06E−047
	X	X	5093	222352151	37.50	poly(rC)-binding protein 1	1.98E−036
	X	X	220988	34740329	39.59	heterogeneous nuclear ribonucleoprotein A3	2.26E−007
	X	X	8683	4506903	25.54	serine/arginine-rich splicing factor 9	4.98E−004
	X	X	5094	14141166	38.22	poly(rC)-binding protein 2 isoform b	2.55E−013
	X	X	1207	4502891	26.22	methylosome subunit pICln	8.07E−012
	X	X	6426	5902076	27.74	serine/arginine-rich splicing factor 1 isoform 1	1.15E−004
	X		4841	34932414	54.23	non-POU domain-containing octamer-binding protein isoform 1	4.44E−006
	X		8570	154355000	73.12	far upstream element-binding protein 2	4.85E−014
X			1984	4503545	16.83	eukaryotic translation initiation factor 5A-1 isoform B	0.00E+000
X			3692	4504771	26.60	eukaryotic translation initiation factor 6 isoform a	4.05E−013
X			2617	1.17E+08	83.16	glycyl-tRNA synthetase	1.89E−018
X			1975	50053795	69.15	eukaryotic translation initiation factor 4B	2.84E−018
X			1938	4503483	95.34	elongation factor 2	1.42E−102
X			23708	46094014	68.88	eukaryotic peptide chain release factor GTP-binding subunit ERF3B	1.19E−004
X			6176	4506669	11.51	60S acidic ribosomal protein P1 isoform 1	5.60E−010
X			6206	14277700	14.51	40S ribosomal protein S12	1.29E−010
X			1937	4503481	50.12	elongation factor 1-gamma	9.40E−016
X			3315	4504517	22.78	heat shock protein beta-1	0.00E+000
X			136319	21956645	12.89	myotrophin	5.60E−018
X			1933	4503477	24.76	elongation factor 1-beta	6.79E−010

† The adjusted pValue assigned to the biological process by the WebGestalt GO analysis using the hypergeometric statistical test with a BH multiple test adjustment.

### Consensus Sequence Analysis

**Figure 2:**
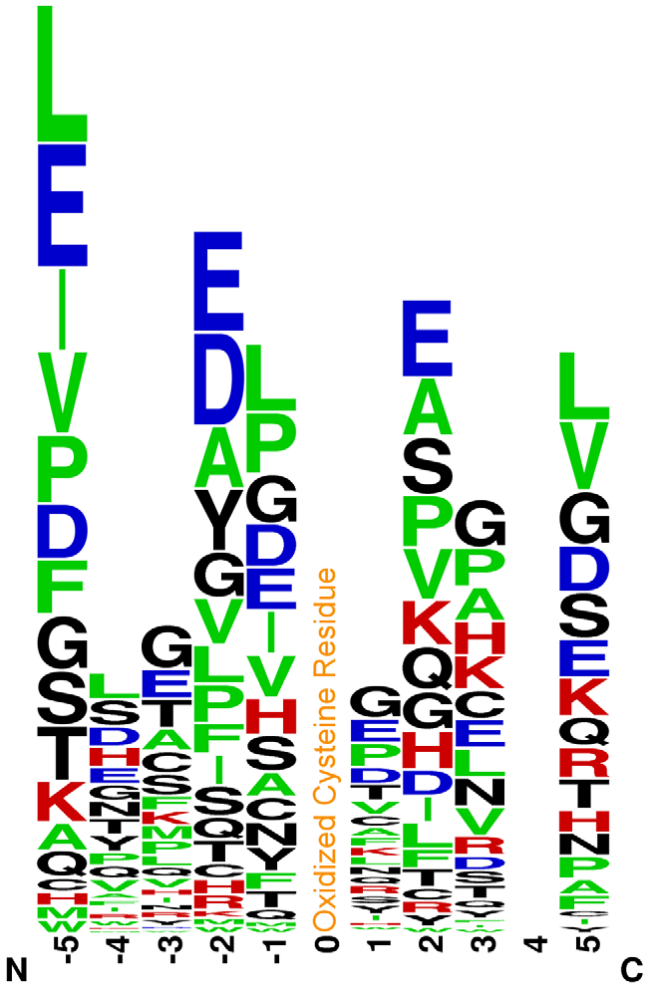
Potential Consensus Sequence for Oxidized Cysteine Residues. The sequence logo representation of the frequency of the amino acid residues surrounding the oxidized cysteine residues, located at position “0”, that were identified from the PROP proteomics procedure.

Using a 1.00e−03 OMSSA eValue confidence cutoff, 78 out of the 115 unique peptides identified with a CAM modification were submitted to the WebLogo [27] online analysis tool as a means of identifying potential consensus sequences associated with cysteine residue oxidation. Figure 2 presents the sequence logo representation that was generated for the frequency of the amino acid residues surrounding the oxidized cysteine residue located at position “0”. Perhaps the most striking feature is the strong prevalence of negatively charged (D and E) and hydrophobic (L, I, V, A) amino acids at the -5, -2, -1, 2, and 5 positions. Note that a negatively charged surface would likely lower the sulfhydryl pK_a_ of the pre-oxidized cysteine residue and, thereby, assist in the initial sulfhydryl deprotonation necessary for its subsequent oxidation [28], [29], [30].

## Discussion

The PROP protocol represents an improved method of identifying proteins and their specific cysteine residues that are oxidized within complex mixtures. While the strategy of blocking uninvolved thiols to enable identification of oxidized sites is not unique, PROP affords some significant enhancements that result in improved performance. First, the rapid fixation of protein samples in TCA prevents artifactual oxidation events. This is facile in tissue culture cells, but should also be possible in intact tissues using immersion or perfusion fixation. Second, the NEM blocking agent is inexpensive and relatively safe. Third, pilot experiments showed that the NEM blockade in guanidine is more efficient than in other denaturing agents, specifically SDS (not shown, though see Templeton, *et al.* [15]).

The guanidine denaturant is problematical when considering its removal prior to subsequent analysis. Procedures such as dialysis and size exclusion chromatography have led in our hands to issues with protein precipitation, a common problem in the removal of many denaturants including 8 M urea. Thus, we focused on deliberate methods of protein precipitation, specifically methanol. Fortunately, methanol is both an effective protein precipitant and also efficiently solubilizes NEM, DTT, guanidine and their reactants. However, proteins precipitated from either dialysis or methanol are soluble later only in denaturing buffers containing SDS or guanidine. This means that the subsequent steps, during which the free thiols or free thiols labeled with molecular tags (e.g. biotin) are captured, must be compatible with denaturants. The biotin-avidin pair used in several other approaches is not compatible with strong denaturants (i.e. guanidine above 2 M). In contrast, the disulfide bonds created between the activated thiol affinity beads and the reduced cysteine residues of proteins used in our approach proceeds efficiently even in buffers with 6 M guanidine. The subsequent release from the thiol beads with simple thiol reducing agents, such as DTT, and labeling with iodoacetamide (IAA) is also advantageous for subsequent mass spectroscopic sequencing approaches. This commonly used reagent pair results in familiar fragmentation patterns simplifying automated and manual sequencing of peptides containing carboxamidomethylated cysteine thiols. In comparison, thiol labeling with moieties containing biotin (e.g. ICAT) can be problematic for sequencing because of their complex fragmentation pattern. Thus, the guanidine denaturant paired with the thiol affinity beads provide an efficient and effective means of enriching for proteins with biologically oxidized cysteine residues using inexpensive, widely available reagents that are well accepted as compatible with downstream mass spectrometry analyses.

The type of proteins quantified using PROP suggests that the protocol has good efficiency. Evidence for that includes the observation that the relatively abundant redox proteins of the family protein disulfide isomerases (PDIs) were matched with 380 scans (including all control and oxidized runs) while other proteins in the cell were identified far less frequently (e.g. actins (274), tubulin (33), histone (0)) with respect to their relative cellular abundance. Further, some proteins were identified that are likely to be of low abundance, for example hepatoma derived growth factor (85 scans, in the oxidized prep only), prostaglandin E synthase 3 (10 scans, in the oxidized prep only) and several protein phosphatases. Thus, the non-specific background of PROP-proteomics appears to be low enough to allow detection of low to moderate abundance redox targets that participate in signal transduction.

We anticipated that PROP would identify many more proteins in the oxidized cell sample than in the control. In fact, the number of unique proteins identified using the 3% FDR cutoff was essentially identical: an average of 94 from both the control and the oxidized samples. However, as illustrated by the list in Table 1, a number of proteins were preferentially identified in the oxidized samples. For example, the small redox molecule thioredoxin was identified in 188 of 203 scans from the oxidized samples, the elongation factor 2 was observed 65 of 69 scans and nucleophosmin was found 228 times exclusively in the oxidized samples.

As stated above, we were surprised to find that some proteins identified from the control sample were not identified in the H_2_O_2_ treated sample. Notably, peroxiredoxin 6, an atypical single-cysteine residue member of the peroxiredoxin family, was identified 14 times from 3 unique, unrelated peptides in the control sample and only once in the oxidized sample. A similar pattern was seen for other proteins recovered preferentially in the control sample, for example a putative serine protease (36 of 36 total scans), a calnexin precursor (16 of 17 total scans) and a basigin precursor (20 of 21 total scans).

The chemical nature of the protein modifications in this category is uncertain. In the basal state, these must have oxidized thiol groups that can be reversed with DTT, most likely S-thiolation, sulfenic acid, or nitrosylation. Following exposure of the cells to H_2_O_2_, these oxidation events must be converted to non-reversible thiol modifications, potentially sulfinic or sulfonic forms. This is perhaps best demonstrated in the case of peroxiredoxins, such as the peroxiredoxin 6, discussed above. Hydrogen peroxide converts active thiols in peroxiredoxins to sulfinic acid [31], [32] that is not reversible by DTT reduction and would, therefore, be predicted to not be identified in PROP from H_2_O_2_ treated cells. Dissection of the nature of these modifications from identified proteins will require individual study.

One strong point of the PROP procedure is the ability to identify the site of *in vivo* protein oxidation through the identification of the CAM modification, rather than the NEM modification, on specific cysteine residues. While we identified many sites of protein oxidation with the experimental design presented herein, we also recognize that we have not fully exploited this feature of the procedure. We envision that, in future experiments, the precipitated proteins could be trypsinized while still attached to the thiopropyl sepharose beads through their target cysteine disulfide linkage(s). Additional washes would then release the non-conjugated peptides and, thereby, further enrich for the specific cysteine targets of oxidation in cells. By significantly reducing the complexity of the peptide mixture that is analyzed, the statistics and accuracy of quantifying oxidation at particular sites and under specific experimental conditions could be greatly enhanced. Furthermore, it would likely result in the identification of peptides from proteins at a lower relative abundance. In future targeted experiments, we intend to approach the problem using both methods.

Some provocative hypotheses can be derived from the identification of the biologically oxidized sites that were identified. For example, galectin 1 is a soluble galactose-binding lectin that is expressed widely during embryogenesis. Regulation of galectin is known to be through intramolecular oxidation, and a non-functional, fully reduced form exists in cells as a non-sugar binding homodimer. In our studies, the carboxamidomethyl peptide _50_ FNAHGDANTIVcNSK_64_ from galectin 1 was observed a total of 6 times in 5 of the 6 H_2_O_2_ treated samples (see Supplemental Data); furthermore, galectin 1 peptides were observed a total of 38 times from 4 unique peptides and only from the 6 oxidized samples. Importantly, Yamaoka, *et al.* [33] identified a uniquely oxidized form of galectin 1, termed t-galectin 1, from NRK cells transformed with the src oncogene (see Table 1). This t-galectin 1 does not bind sugars but is a potent mitogen of NIH 3T3 cells. Our identification of this oxidized residue does not prove that peroxide stimulated HeLa cells may begin to make this mitogenic form, but the PROP procedure does enable the identification and surveillance of these unique protein modifications.

As another example, cofilin 1 is an actin binding protein that enhances actin polymerization. Treatment of T cells with hydrogen peroxide is accompanied by a blockade to T cell activation that has been tied to the oxidation status of cofilin [34]. Using *purified* cofilin treated with hydrogen peroxide *in vitro*, this group used mass spectrometry to identify sulfonic oxidation of cysteine at residue C139. Our work identified the reversible oxidation of C139 (10 scans) together with the NEM labeled version of both C139 (5 scans) and C39 (20 scans), suggesting the presence of at least one additional oxidation site to explain the presence of the NEM-labeled C39 peptide in complexes washed with denaturing buffer. Through analysis of mutants, the cited work did demonstrate that C39 was essential to the biological function of cofilin. Regardless, this further demonstrates that PROP-proteomics is able to observe biologically relevant oxidation of target proteins in intact cells.

In summary, we have demonstrated the use of the PROP protocol to purify and subsequently analyze proteins with reversible oxidation of cysteine residues using mass spectrometry. Using an optimized system that (1) employs acid quenching of samples to prevent artifacts, (2) efficiently removes non-oxidized cysteine thiol targets, and (3) generates peptides with cysteine residues in a modified state conducive to mass spectral sequence identification, this PROP proteomics methodology offers the chance to identify proteins and their specific targets of cysteine thiol oxidation.

## Supporting Information

Script S1 **VBA Script for Protein Summary.** This document includes the Excel macro, written in Excel 2007, that was used to generate the spreadsheet “PROP_Proteomics_Summary.xlsx” that represents the Table S1 of the Supporting Information.
(DOC)

Table S1 **Summary of Mass Spectral Search Results.** An Excel spreadsheet containing a list of the peptide sequence matches identified using the OMSSA search engine. Separate worksheets list the peptide matches that passed the FDR filter; the unique peptides from the FDR-filtered matches; the unique proteins from the FDR-filtered matches; and the complete data used to generate the results presented in Tables 1–5 of the manuscript.
(XLSX)

Analysis S1 **WebGestalt Results.** This document contains the gene ontology (GO), KEGG enrichment and Wikipathways gene enrichment analysis obtained through using the web-based gene set analysis package WebGestalt [26].
(DOC)

## References

[1] Zheng M, Aslund F, Storz G (1998). Activation of the OxyR transcription factor by reversible disulfide bond formation.. Science.

[2] Cross JV, Templeton DJ (2004). Oxidative stress inhibits MEKK1 by site-specific glutathionylation in the ATP-binding domain.. Biochem J.

[3] Cross JV, Templeton DJ (2006). Regulation of signal transduction through protein cysteine oxidation.. Antioxid Redox Signal.

[4] Chu F, Ward NE, O'Brian CA (2003). PKC isozyme S-cysteinylation by cystine stimulates the pro-apoptotic isozyme PKC delta and inactivates the oncogenic isozyme PKC epsilon.. Carcinogenesis.

[5] Nadeau PJ, Charette SJ, Landry J (2009). REDOX reaction at ASK1-Cys250 is essential for activation of JNK and induction of apoptosis.. Mol Biol Cell.

[6] Wani R, Qian J, Yin L, Bechtold E, King SB (2011). Isoform-specific regulation of Akt by PDGF-induced reactive oxygen species.. Proc Natl Acad Sci U S A.

[7] Boivin B, Zhang S, Arbiser JL, Zhang ZY, Tonks NK (2008). A modified cysteinyl-labeling assay reveals reversible oxidation of protein tyrosine phosphatases in angiomyolipoma cells.. Proc Natl Acad Sci U S A.

[8] Klomsiri C, Nelson KJ, Bechtold E, Soito L, Johnson LC (2010). Use of dimedone-based chemical probes for sulfenic acid detection evaluation of conditions affecting probe incorporation into redox-sensitive proteins.. Methods in Enzymology.

[9] Sethuraman M, McComb ME, Heibeck T, Costello CE, Cohen RA (2004). Isotope-coded affinity tag approach to identify and quantify oxidant-sensitive protein thiols.. Mol Cell Proteomics.

[10] Sethuraman M, McComb ME, Huang H, Huang S, Heibeck T (2004). Isotope-coded affinity tag (ICAT) approach to redox proteomics: identification and quantitation of oxidant-sensitive cysteine thiols in complex protein mixtures.. J Proteome Res.

[11] Fu C, Wu C, Liu T, Ago T, Zhai P (2009). Elucidation of thioredoxin target protein networks in mouse.. Mol Cell Proteomics.

[12] Hagglund P, Bunkenborg J, Maeda K, Svensson B (2008). Identification of thioredoxin disulfide targets using a quantitative proteomics approach based on isotope-coded affinity tags.. J Proteome Res.

[13] Zander T, Phadke ND, Bardwell JC (1998). Disulfide bond catalysts in Escherichia coli.. Methods in Enzymology.

[14] Bardwell J (2005). Thiol modifications in a snapshot.. Nature Biotechnology.

[15] Templeton DJ, Aye MS, Rady J, Xu F, Cross JV (2010). Purification of reversibly oxidized proteins (PROP) reveals a redox switch controlling p38 MAP kinase activity.. PLoS One.

[16] Held JM, Danielson SR, Behring JB, Atsriku C, Britton DJ (2010). Targeted quantitation of site-specific cysteine oxidation in endogenous proteins using a differential alkylation and multiple reaction monitoring mass spectrometry approach.. Mol Cell Proteomics.

[17] Lyons CE, Victor KG, Moshnikov SA, Bachmann LM, Baras AS (2011). PICquant: A Quantitative Platform To Measure Differential Peptide Abundance Using Dual-Isotopic Labeling with 12C6- and 13C6-Phenyl Isocyanate.. Analytical Chemistry.

[18] Victor KG, Murgai M, Lyons CE, Templeton TA, Moshnikov SA (2010). MAZIE: A Mass and Charge Inference Engine to Enhance Database Searching of Tandem Mass Spectra.. Journal of the American Society for Mass Spectrometry.

[19] Geer LY, Markey SP, Kowalak JA, Wagner L, Xu M (2004). Open mass spectrometry search algorithm.. Journal of Proteome Research.

[20] Kall L, Storey JD, MacCoss MJ, Noble WS (2008). Assigning significance to peptides identified by tandem mass spectrometry using decoy databases.. Journal of Proteome Research.

[21] Liu HB, Sadygov RG, Yates JR (2004). A model for random sampling and estimation of relative protein abundance in shotgun proteomics.. Analytical Chemistry.

[22] Hendrickson EL, Xia QW, Wang TS, Leigh JA, Hackett M (2006). Comparison of spectral counting and metabolic stable isotope labeling for use with quantitative microbial proteomics.. Analyst.

[23] Old WM, Meyer-Arendt K, Aveline-Wolf L, Pierce KG, Mendoza A (2005). Comparison of label-free methods for quantifying human proteins by shotgun proteomics.. Molecular & Cellular Proteomics.

[24] Usaite R, Wohlschlegel J, Venable JD, Park SK, Nielsen J (2008). Characterization of global yeast quantitative proteome data generated from the wild-type and glucose repression Saccharomyces cerevisiae strains: The comparison of two quantitative methods.. Journal of Proteome Research.

[25] Collier TS, Sarkar P, Franck WL, Rao BM, Dean RA (2010). Direct Comparison of Stable Isotope Labeling by Amino Acids in Cell Culture and Spectral Counting for Quantitative Proteomics.. Analytical Chemistry.

[26] Zhang B, Kirov S, Snoddy J (2005). WebGestalt: an integrated system for exploring gene sets in various biological contexts.. Nucleic Acids Research.

[27] Crooks GE, Hon G, Chandonia JM, Brenner SE (2004). WebLogo: a sequence logo generator.. Genome Research.

[28] Denu JM, Tanner KG (1998). Specific and reversible inactivation of protein tyrosine phosphatases by hydrogen peroxide: evidence for a sulfenic acid intermediate and implications for redox regulation.. Biochemistry.

[29] Wood ZA, Schroder E, Robin Harris J, Poole LB (2003). Structure, mechanism and regulation of peroxiredoxins.. Trends in Biochemical Sciences.

[30] Salsbury FR, Knutson ST, Poole LB, Fetrow JS (2008). Functional site profiling and electrostatic analysis of cysteines modifiable to cysteine sulfenic acid.. Protein Sci.

[31] Woo HA, Chae HZ, Hwang SC, Yang KS, Kang SW (2003). Reversing the inactivation of peroxiredoxins caused by cysteine sulfinic acid formation.. Science.

[32] Wood ZA, Poole LB, Karplus PA (2003). Peroxiredoxin evolution and the regulation of hydrogen peroxide signaling.. Science.

[33] Yamaoka K, Ingendoh A, Tsubuki S, Nagai Y, Sanai Y (1996). Structural and functional characterization of a novel tumor-derived rat galectin-1 having transforming growth factor (TGF) activity: the relationship between intramolecular disulfide bridges and TGF activity.. J Biochem.

[34] Klemke M, Wabnitz GH, Funke F, Funk B, Kirchgessner H (2008). Oxidation of cofilin mediates T cell hyporesponsiveness under oxidative stress conditions.. Immunity.

