# Supplementary material for: Proteomic Profile of Reversible Protein Oxidation Using PROP, Purification of Reversibly Oxidized Proteins
Source: PLoS One. 2012 Feb 28;7(2):e32527. doi: 10.1371/journal.pone.0032527 (PMC3289665; doi:10.1371/journal.pone.0032527)
Supplement: Script S1 — VBA Script for Protein Summary. This document includes the Excel macro, written in Excel 2007, that was used to generate the spreadsheet “PROP_Proteomics_Summary.xlsx” that represents the Table S1 of the Supporting Information. (DOC) [file pone.0032527.s001.doc]

This document includes the Excel macro, written in Excel 2007, that was used to generate the spreadsheet “Table S1 - PROP_Proteomics_Summary.xlsx” that is contained in the Supporting Information. The macro asks the user to indicate whether to merge or compare the search results that are to be combined. The “merge” option is used to combine two search (or more) results from the same mass spec data (run) while the “compare option is used to combine two (or more) search results from different mass spec data (runs). The script then asks the user to input how many files are to be combined in this manner. Next the script has the user pick each of these data files by use of the standard “Browse” window. The script as it this current version assumes that these data files were generated by OMSSA search engine from the line command option with flag “-oc”. However, the script can be readily modified for other search result formats as long as the scan number, peptide sequence, sequence match score and protein identifier (e.g., gi, ipi, etc.) exist as columns. The results from each data file are placed in a separate worksheet and the scan results are compiled, keeping track of which sample each scan match originated from. The user is then asked to input the FDR cutoff percent that is to be used to determine the peptides and proteins that were confidently identified.

' Declare Public Variables

Public Ri As Long

Public Rlast As Long

Public C_total As Integer

Public C_gi As Integer

Public C_scan As Integer

Public C_seq As Integer

Public Input_FDRfilter As Single

Public SourceSheet As Worksheet

' Main program starts

Sub Results2Combine()

Dim NumToOpen_default As Integer

Dim NumToOpen As Integer

Dim TypeToOpen_default As String

Dim TypeToOpen As String

' Indicates which column the information is located in

C_total = 12

C_gi = 1

C_scan = 2

C_seq = 4

TypeToOpen_default = "compare"

Input_FDRfilter_default = 2

Application.ScreenUpdating = False

Call Results_Get_Type(TypeToOpen, TypeToOpen_default)

If StrComp(TypeToOpen, "merge") = 0 Then

NumToOpen_default = 2

ElseIf StrComp(TypeToOpen, "compare") = 0 Then

NumToOpen_default = 2

End If

Call Results_Get_NumToOpen(NumToOpen, NumToOpen_default)

Call Results_FilesOpen(TypeToOpen, NumToOpen, C_total)

Call Results_Combine(NumToOpen, C_total)

If StrComp(TypeToOpen, "merge") = 0 Then

Call Results_Merge_Scans(NumToOpen, C_total, C_scan)

ElseIf StrComp(TypeToOpen, "compare") = 0 Then

Call Results_Compare_Scans(NumToOpen, C_total, C_scan)

End If

Call User_Input_FDR(Input_FDRfilter_default)

Call Results_Combine_Scans_FDR(NumToOpen, C_total, C_scan)

Call Results_Combine_Peptide(NumToOpen, C_total, C_seq)

Call Results_Format_Peptide(NumToOpen, C_total, C_gi)

Call Results_Combine_Protein(NumToOpen, C_gi)

Call Results_Format_Protein(NumToOpen, C_total, C_gi)

Application.ScreenUpdating = True

End Sub

' Lets user choose whether to merge or compare the search results

' Use merge to combine two search (or more) results from the same mass spec data (run)

' Use “compare” to combine two (or more) search results from different mass spec data (runs)

Private Sub Results_Get_Type(TypeToOpen, TypeToOpen_default)

Dim TypeToOpen_check As Integer

TypeToOpen_check = 0

TypeToOpen = TypeToOpen_default

Do While TypeToOpen_check = 0

TypeToOpen = InputBox("Please enter 'merge' scans or 'compare' peptides:", "Type of File Merge", TypeToOpen_default)

If (StrComp(TypeToOpen, "merge") = 0) Or (StrComp(TypeToOpen, "compare") = 0) Then

TypeToOpen_check = 1

End If

Loop

End Sub

' User inputs how many mass spec data files are to be combined

Private Sub Results_Get_NumToOpen(NumToOpen, NumToOpen_default)

Dim NumToOpen_check As Integer

NumToOpen_check = 0

NumToOpen = NumToOpen_default

Do While NumToOpen_check = 0

NumToOpen = InputBox("Please enter the Number of Samples to combine:", "Number of Files:", NumToOpen_default)

If IsNumeric(NumToOpen) Then

NumToOpen = CInt(NumToOpen)

If NumToOpen <= 20 Then

If NumToOpen > 1 Then

NumToOpen_check = 1

End If

End If

End If

Loop

End Sub

' Opens mass data files chosen by user through a “browse” window interface

Private Sub Results_FilesOpen(TypeToOpen, NumToOpen, C_total)

Dim NumToOpen_loop As Integer

Dim fileToOpen_flag As Integer

Dim Counter As Integer

' Create new file for Comparison Summary ".xls" document

Original_sheet_count = Application.SheetsInNewWorkbook

Application.SheetsInNewWorkbook = NumToOpen + 3

Set Summary_WorkBook = Workbooks.Add

Application.SheetsInNewWorkbook = Original_sheet_count

Set Summary_WorkBook = Nothing

Workbook_name = ActiveWorkbook.Name

Sheets(NumToOpen + 1).Select

ActiveSheet.Name = "Combined Scans"

Set SourceSheet = Worksheets("Combined Scans")

Sheets(NumToOpen + 2).Select

ActiveSheet.Name = "Peptide Summary"

Sheets(NumToOpen + 3).Select

ActiveSheet.Name = "Protein Summary"

Application.ScreenUpdating = True

Application.DisplayAlerts = False

For NumToOpen_loop = 1 To NumToOpen

fileToOpen_flag = 0

Do While fileToOpen_flag = 0

fileToOpen = Application.GetOpenFilename("Excel Files (*.xls*), *.xls*")

If fileToOpen <> False Then

fileToOpen_flag = 1

Workbooks.Open (fileToOpen)

data_workbook_name = ActiveWorkbook.Name

data_sheet_name = Left(data_workbook_name, Len(data_workbook_name) - 5)

If Len(data_sheet_name) > 31 Then

data_sheet_name = Right(data_sheet_name, 31)

End If

Sheets("Scan Summary").Select

Cells.Copy

Workbooks(Workbook_name).Activate

Sheets(NumToOpen_loop).Select

ActiveSheet.Paste

ActiveSheet.Name = data_sheet_name

Range("A1").Select

Workbooks(data_workbook_name).Close SaveChanges:=False

End If

Loop

Application.ScreenUpdating = True

Application.ScreenUpdating = False

Next NumToOpen_loop

'Copy the header from the scan summary of the first individual file

Sheets(1).Select

Counter = 1

While (Not (StrComp(Sheets(1).Cells(1, Counter).Value, "gi", 1) = 0)) And (Counter < 25)

Counter = Counter + 1

Wend

Range(Cells(1, Counter), Cells(1, Counter + C_total - 1)).Copy Destination:=Sheets("Combined Scans").Range("A1")

Range("A1").Select

Application.DisplayAlerts = True

End Sub

' Combines all of the scans from all of the user-chosen mass spec data

Private Sub Results_Combine(NumToOpen, C_total)

Dim Rfirst As Long

Dim Rnext As Integer

Dim NumToOpen_loop As Integer

Dim C_start As Integer

Rfirst = 2

Rlast = 1

For NumToOpen_loop = 1 To NumToOpen

Sheets(NumToOpen_loop).Select

C_start = 1

While (Not (StrComp(Sheets(1).Cells(1, C_start).Value, "gi", 1) = 0)) And (C_start < 25)

C_start = C_start + 1

Wend

Rnext = Range(Cells(2, C_start), Cells(2, C_start).End(xlDown)).Rows.Count

Rlast = Rlast + Rnext

Range(Cells(2, C_start).End(xlDown), Cells(2, C_start + C_total - 1)).Select

Selection.Copy

SourceSheet.Select

Range(Cells(Rfirst, NumToOpen_loop), Cells(Rfirst + Rnext, NumToOpen_loop + C_total - 1)).Select

ActiveSheet.Paste

Columns(NumToOpen_loop).Select

Selection.Insert Shift:=xlToRight

Cells(1, NumToOpen_loop).Select

ActiveCell.FormulaR1C1 = NumToOpen_loop

Cells(Rfirst, NumToOpen_loop).FormulaR1C1 = "X"

Range(Cells(Rfirst, NumToOpen_loop), Cells(Rlast, NumToOpen_loop)).Select

Selection.FillDown

Sheets(NumToOpen_loop).Select

Range("A1").Select

Rfirst = Rlast + 1

Next NumToOpen_loop

End Sub

' Merges mass spec data results by assigning the search result with the lowest (best) Evalue to each scan number

Private Sub Results_Merge_Scans(NumToOpen, C_total, C_scan)

Dim Rfirst As Integer

Dim Rsummary As Integer

Dim NumToOpen_loop As Integer

Worksheets.Add(After:=SourceSheet).Name = "Scan Summary"

SourceSheet.Select

'Move Sample Tally to end of columns

Range(Cells(1, 1), Cells(1, NumToOpen + 1).End(xlDown).Offset(0, -1)).Select

Selection.Cut

Columns(NumToOpen + C_total + 1).Select

Selection.Insert Shift:=xlToRight

'Count number of total scans

Rlast = Range(Cells(1, 1), Cells(1, 1).End(xlDown)).Rows.Count

'Sort by Scan Number and then Evalue

ActiveWorkbook.Worksheets("Combined Scans").Sort.SortFields.Clear

ActiveWorkbook.Worksheets("Combined Scans").Sort.SortFields.Add Key:=Range("B2"), SortOn:=xlSortOnValues, Order:=xlAscending, DataOption:=xlSortNormal

ActiveWorkbook.Worksheets("Combined Scans").Sort.SortFields.Add Key:=Range("C2"), SortOn:=xlSortOnValues, Order:=xlAscending, DataOption:=xlSortNormal

With ActiveWorkbook.Worksheets("Combined Scans").Sort

.SetRange Range(Cells(1, 1), Cells(Rlast, C_total + NumToOpen))

.Header = xlYes

.MatchCase = False

.Orientation = xlTopToBottom

.SortMethod = xlPinYin

.Apply

End With

Call Results_Format_Scans

'Move Sample Tally back to the front of columns

For NumToOpen_loop = 1 To NumToOpen

Columns(C_total + NumToOpen_loop).Select

Selection.Cut

Columns(NumToOpen_loop).Select

Selection.Insert Shift:=xlToRight

Next NumToOpen_loop

Call Results_Format_Col(NumToOpen)

'Merge scans and put results in "Scan Summary" sheet

SourceSheet.Rows(1).Copy Destination:=Worksheets("Scan Summary").Rows(1) 'Copy Header Row

Rfirst = 2

Rsummary = 2

SourceSheet.Rows(Rfirst).Copy Destination:=Worksheets("Scan Summary").Rows(Rsummary) 'Copy 1st Data Row

For Ri = 3 To Rlast

If StrComp(SourceSheet.Cells(Rfirst, NumToOpen + C_scan).Value, SourceSheet.Cells(Ri, NumToOpen + C_scan).Value, 1) = 0 Then

For NumToOpen_loop = 1 To NumToOpen

If StrComp(SourceSheet.Cells(Ri, NumToOpen_loop).Value, "X", 1) = 0 Then

SourceSheet.Cells(Ri, NumToOpen_loop).Copy Destination:=Worksheets("Scan Summary").Cells(Rsummary, NumToOpen_loop)

End If

Next NumToOpen_loop

Else

Rfirst = Ri

Rsummary = Rsummary + 1

SourceSheet.Rows(Rfirst).Copy Destination:=Worksheets("Scan Summary").Rows(Rsummary)

End If

Next Ri

Set SourceSheet = Worksheets("Scan Summary")

End Sub

' For comparsion of data from two (or more) different mass spec datafiles, sorts by scan number

Private Sub Results_Compare_Scans(NumToOpen, C_total, C_scan)

Dim Rfirst As Integer

Dim Rsummary As Integer

Dim NumToOpen_loop As Integer

SourceSheet.Select

ActiveSheet.Name = "Scan Summary"

'Move Sample Tally to end of columns

Range(Cells(1, 1), Cells(1, NumToOpen + 1).End(xlDown).Offset(0, -1)).Select

Selection.Cut

Columns(NumToOpen + C_total + 1).Select

Selection.Insert Shift:=xlToRight

Cells.Select

Selection.Sort Key1:=Range("B2"), Order1:=xlAscending, Header:=xlYes, OrderCustom:=1, MatchCase:=False, Orientation:=xlTopToBottom, DataOption1:=xlSortNormal

Call Results_Format_Scans

For NumToOpen_loop = 1 To NumToOpen

Columns(C_total + NumToOpen_loop).Select

Selection.Cut

Columns(NumToOpen_loop).Select

Selection.Insert Shift:=xlToRight

Next NumToOpen_loop

Call Results_Format_Col(NumToOpen)

Set SourceSheet = Worksheets("Scan Summary")

End Sub

' Ask for user in input the FDR rate to be used as the cutoff

Private Sub User_Input_FDR(Input_FDRfilter_default)

Dim Input_FDRfilter_check As Integer

Input_FDRfilter_check = 0

Input_FDRfilter = Input_FDRfilter_default

Do While Input_FDRfilter_check = 0

Input_FDRfilter = InputBox("Please enter the FDR filter Cutoff as percent:", "FDR Filter (%):", Input_FDRfilter_default)

If IsNumeric(Input_FDRfilter) Then

If CDbl(Input_FDRfilter) <= 10 Then

Input_FDRfilter_check = 1

End If

End If

Loop

Input_FDRfilter = Input_FDRfilter / 100

End Sub

' Calculates the FDR for each scan according to its Evalue and then, for all that pass the user-defined FDR cutoff, copies the scan info to ”Unique FDR Filtered Scans” sheet

Private Sub Results_Combine_Scans_FDR(NumToOpen, C_total, C_scan)

Dim Rlast As Long

Worksheets.Add(After:=SourceSheet).Name = "Unique FDR Filtered Scans"

SourceSheet.Select

'Move Sample Tally to end of columns

Range(Cells(1, 1), Cells(1, NumToOpen + 1).End(xlDown).Offset(0, -1)).Select

Selection.Cut

Columns(NumToOpen + C_total + 1).Select

Selection.Insert Shift:=xlToRight

'Sort by Evalue

Cells.Select

Selection.Sort Key1:=Range("C2"), Order1:=xlAscending, Header:=xlYes, OrderCustom:=1, MatchCase:=False, Orientation:=xlTopToBottom, DataOption1:=xlSortNormal

Cells(2, C_total + NumToOpen + 1).FormulaR1C1 = "=EXACT(RC[-" & C_total + NumToOpen & "],0)"

Cells(2, C_total + NumToOpen + 2).FormulaR1C1 = "=COUNTIF(R2C[-1]:RC[-1], ""TRUE"")/COUNTIF(R2C[-1]:RC[-1], ""FALSE"")"

Cells(2, C_total + NumToOpen + 3).FormulaR1C1 = "=COUNTIF(R2C[-2]:RC[-2], ""FALSE"") - COUNTIF(R2C[-2]:RC[-2], ""TRUE"")"

Rlast = Range(Cells(1, 1), Cells(1, 1).End(xlDown)).Rows.Count

Range(Cells(2, C_total + NumToOpen + 1), Cells(Rlast, C_total + NumToOpen + 3)).Select

Selection.FillDown

Range(Cells(1, C_total + NumToOpen + 2), Cells(1, C_total + NumToOpen + 3)).Select

With Selection.Interior

.ColorIndex = 39

.Pattern = xlSolid

End With

With Selection

.HorizontalAlignment = xlCenter

.VerticalAlignment = xlCenter

.WrapText = True

.Orientation = 0

.ShrinkToFit = False

.MergeCells = False

End With

Selection.NumberFormat = "@"

With Selection.Font

.Name = "Arial"

.FontStyle = "Bold"

.Size = 10

End With

Cells(1, C_total + NumToOpen + 2).FormulaR1C1 = "FDR"

Cells(1, C_total + NumToOpen + 3).FormulaR1C1 = "True Positives"

Range(Cells(2, C_total + NumToOpen + 2), Cells(Rlast, C_total + NumToOpen + 2)).Select

Selection.NumberFormat = "0.000"

Selection.FormatConditions.Delete

Selection.FormatConditions.Add Type:=xlCellValue, Operator:=xlLess, Formula1:=Input_FDRfilter

Selection.FormatConditions(1).Interior.ColorIndex = 35

Range(Cells(2, C_total + NumToOpen + 3), Cells(Rlast, C_total + NumToOpen + 3)).Select

Selection.NumberFormat = "0"

Selection.FormatConditions.Delete

Call Results_Format_Scans

For NumToOpen_loop = 1 To NumToOpen

Columns(C_total + NumToOpen_loop).Select

Selection.Cut

Columns(NumToOpen_loop).Select

Selection.Insert Shift:=xlToRight

Next NumToOpen_loop

Call Results_Format_Col(NumToOpen)

For Ri = Rlast To 2 Step -1

If Cells(Ri, C_total + NumToOpen + 2).Value < CDbl(Input_FDRfilter) Then Exit For

Next Ri

Range(Cells(1, 1), Cells(Ri, C_total + NumToOpen)).Copy

Sheets("Unique FDR Filtered Scans").Select

ActiveSheet.Paste

Application.CutCopyMode = False

Range(Cells(1, 1), Cells(1, NumToOpen + 1).End(xlDown).Offset(0, -1)).Select

Selection.Cut

Columns(NumToOpen + C_total + 1).Select

Selection.Insert Shift:=xlToRight

Call Results_Format_Scans

For NumToOpen_loop = 1 To NumToOpen

Columns(C_total + NumToOpen_loop).Select

Selection.Cut

Columns(NumToOpen_loop).Select

Selection.Insert Shift:=xlToRight

Next NumToOpen_loop

Call Results_Format_Col(NumToOpen)

Set SourceSheet = Worksheets("Unique FDR Filtered Scans")

End Sub

' Consolidates the scans that passed the user-defined FDR cutoff to create a summary of the unique peptides, keeping track of from which datafile each peptide was identified

Private Sub Results_Combine_Peptide(NumToOpen, C_total, C_seq)

Dim Rfirst As Integer

Dim Rsummary As Integer

Dim NumToOpen_loop As Integer

SourceSheet.Select

'Move Sample Tally to end of columns

Range(Cells(1, 1), Cells(1, NumToOpen + 1).End(xlDown).Offset(0, -1)).Select

Selection.Cut

Columns(NumToOpen + C_total + 1).Select

Selection.Insert Shift:=xlToRight

'Count number of total scans

Rlast = Range(Cells(1, 1), Cells(1, 1).End(xlDown)).Rows.Count

'Sort by Scan Number and then Evalue

ActiveWorkbook.Worksheets("Unique FDR Filtered Scans").Sort.SortFields.Clear

ActiveWorkbook.Worksheets("Unique FDR Filtered Scans").Sort.SortFields.Add Key:=Range("D2"), SortOn:=xlSortOnValues, Order:=xlAscending, DataOption:=xlSortNormal

ActiveWorkbook.Worksheets("Unique FDR Filtered Scans").Sort.SortFields.Add Key:=Range("C2"), SortOn:=xlSortOnValues, Order:=xlAscending, DataOption:=xlSortNormal

With ActiveWorkbook.Worksheets("Unique FDR Filtered Scans").Sort

.SetRange Range(Cells(1, 1), Cells(Rlast, C_total + NumToOpen))

.Header = xlYes

.MatchCase = False

.Orientation = xlTopToBottom

.SortMethod = xlPinYin

.Apply

End With

Call Results_Format_Scans

'Move Sample Tally back to the front of columns

For NumToOpen_loop = 1 To NumToOpen

Columns(C_total + NumToOpen_loop).Select

Selection.Cut

Columns(NumToOpen_loop).Select

Selection.Insert Shift:=xlToRight

Next NumToOpen_loop

Call Results_Format_Col(NumToOpen)

SourceSheet.Rows(1).Copy Destination:=Worksheets("Peptide Summary").Rows(1) 'Copy Header Row

Rfirst = 2

Rsummary = 2

ReDim counter_array(1 To NumToOpen) As Integer

SourceSheet.Rows(Rfirst).Copy Destination:=Worksheets("Peptide Summary").Rows(Rsummary) 'Copy 1st Data Row

For NumToOpen_loop = 1 To NumToOpen

If StrComp(SourceSheet.Cells(Rfirst, NumToOpen_loop).Value, "X", 1) = 0 Then

counter_array(NumToOpen_loop) = counter_array(NumToOpen_loop) + 1

End If

Next NumToOpen_loop

For Ri = 3 To Rlast

If StrComp(SourceSheet.Cells(Rfirst, NumToOpen + C_seq).Value, SourceSheet.Cells(Ri, NumToOpen + C_seq).Value, 1) = 0 Then

For NumToOpen_loop = 1 To NumToOpen

If StrComp(SourceSheet.Cells(Ri, NumToOpen_loop).Value, "X", 1) = 0 Then

counter_array(NumToOpen_loop) = counter_array(NumToOpen_loop) + 1

End If

Next NumToOpen_loop

Else

For NumToOpen_loop = 1 To NumToOpen

Worksheets("Peptide Summary").Cells(Rsummary, NumToOpen_loop).Value = counter_array(NumToOpen_loop)

Next NumToOpen_loop

Rfirst = Ri

Rsummary = Rsummary + 1

ReDim counter_array(1 To NumToOpen) As Integer

SourceSheet.Rows(Rfirst).Copy Destination:=Worksheets("Peptide Summary").Rows(Rsummary)

For NumToOpen_loop = 1 To NumToOpen

If StrComp(SourceSheet.Cells(Ri, NumToOpen_loop).Value, "X", 1) = 0 Then

counter_array(NumToOpen_loop) = counter_array(NumToOpen_loop) + 1

End If

Next NumToOpen_loop

End If

Next Ri

'Output results for last peptide

For NumToOpen_loop = 1 To NumToOpen

Worksheets("Peptide Summary").Cells(Rsummary, NumToOpen_loop).Value = counter_array(NumToOpen_loop)

Next NumToOpen_loop

End Sub

' Consolidates the scans that passed the user-defined FDR cutoff to create a summary of the proteins, keeping track of from which datafile each protein was identified

Private Sub Results_Combine_Protein(NumToOpen, C_gi)

Dim Rfirst As Integer

Dim Rsummary As Integer

Dim NumToOpen_loop As Integer

Sheets("Peptide Summary").Select

'Count number of total scans

Rlast = Range(Cells(1, 1), Cells(1, 1).End(xlDown)).Rows.Count - 1 'the minus 1 accounts for the tabulations at the end of the columns

Worksheets("Peptide Summary").Rows(1).Copy Destination:=Worksheets("Protein Summary").Rows(1) 'Copy Header Row

Rfirst = 2

Rsummary = 2

ReDim total_counter(1 To NumToOpen) As Integer

ReDim unique_counter(1 To NumToOpen) As Integer

Worksheets("Peptide Summary").Rows(Rfirst).Copy Destination:=Worksheets("Protein Summary").Rows(Rsummary) 'Copy 1st Peptide Data Row

For NumToOpen_loop = 1 To NumToOpen

total_counter(NumToOpen_loop) = total_counter(NumToOpen_loop) + Worksheets("Peptide Summary").Cells(Rfirst, NumToOpen_loop).Value

If StrComp(Worksheets("Peptide Summary").Cells(Rfirst, NumToOpen_loop).Value, "0", 1) <> 0 Then

unique_counter(NumToOpen_loop) = unique_counter(NumToOpen_loop) + 1

End If

Next NumToOpen_loop

For Ri = 3 To Rlast

If Worksheets("Peptide Summary").Cells(Rfirst, NumToOpen + C_gi).Value = Worksheets("Peptide Summary").Cells(Ri, NumToOpen + C_gi).Value Then

For NumToOpen_loop = 1 To NumToOpen

total_counter(NumToOpen_loop) = total_counter(NumToOpen_loop) + Worksheets("Peptide Summary").Cells(Ri, NumToOpen_loop).Value

If StrComp(Worksheets("Peptide Summary").Cells(Ri, NumToOpen_loop).Value, "0", 1) <> 0 Then

unique_counter(NumToOpen_loop) = unique_counter(NumToOpen_loop) + 1

End If

Next NumToOpen_loop

Else

For NumToOpen_loop = 1 To NumToOpen

Worksheets("Protein Summary").Cells(Rsummary, NumToOpen_loop).Value = unique_counter(NumToOpen_loop) + total_counter(NumToOpen_loop) / 1000

If (unique_counter(NumToOpen_loop) + total_counter(NumToOpen_loop)) = 0 Then

Worksheets("Protein Summary").Cells(Rsummary, NumToOpen_loop).Value = "X"

End If

Next NumToOpen_loop

Rfirst = Ri

Rsummary = Rsummary + 1

ReDim total_counter(1 To NumToOpen) As Integer

ReDim unique_counter(1 To NumToOpen) As Integer

Worksheets("Peptide Summary").Rows(Rfirst).Copy Destination:=Worksheets("Protein Summary").Rows(Rsummary) 'Copy next Peptide Data Row

For NumToOpen_loop = 1 To NumToOpen

total_counter(NumToOpen_loop) = total_counter(NumToOpen_loop) + Worksheets("Peptide Summary").Cells(Ri, NumToOpen_loop).Value

If StrComp(Worksheets("Peptide Summary").Cells(Ri, NumToOpen_loop).Value, "0", 1) <> 0 Then

unique_counter(NumToOpen_loop) = unique_counter(NumToOpen_loop) + 1

End If

Next NumToOpen_loop

End If

Next Ri

'Output results for last protein

For NumToOpen_loop = 1 To NumToOpen

Worksheets("Protein Summary").Cells(Rsummary, NumToOpen_loop).Value = unique_counter(NumToOpen_loop) + total_counter(NumToOpen_loop) / 1000

If (unique_counter(NumToOpen_loop) + total_counter(NumToOpen_loop)) = 0 Then

Worksheets("Protein Summary").Cells(Rsummary, NumToOpen_loop).Value = "X"

End If

Next NumToOpen_loop

End Sub

' Formats the “Peptide Summary” sheet

Private Sub Results_Format_Peptide(NumToOpen, C_total, C_gi)

Dim delete_check As String

Dim C_cut As Integer

Sheets("Peptide Summary").Select

'Move Sample Tally to end of columns

Range(Cells(1, 1), Cells(1, NumToOpen + 1).End(xlDown).Offset(0, -1)).Select

Selection.Cut

Columns(NumToOpen + C_total + 1).Select

Selection.Insert Shift:=xlToRight

'Count number of total scans

Rlast = Range(Cells(1, 1), Cells(1, 1).End(xlDown)).Rows.Count

'Sort by gi and then peptide

ActiveWorkbook.Worksheets("Peptide Summary").Sort.SortFields.Clear

ActiveWorkbook.Worksheets("Peptide Summary").Sort.SortFields.Add Key:=Range("A2"), SortOn:=xlSortOnValues, Order:=xlAscending, DataOption:=xlSortNormal

ActiveWorkbook.Worksheets("Peptide Summary").Sort.SortFields.Add Key:=Range("C2"), SortOn:=xlSortOnValues, Order:=xlAscending, DataOption:=xlSortNormal

With ActiveWorkbook.Worksheets("Peptide Summary").Sort

.SetRange Range(Cells(1, 1), Cells(Rlast, C_total + NumToOpen))

.Header = xlYes

.MatchCase = False

.Orientation = xlTopToBottom

.SortMethod = xlPinYin

.Apply

End With

Call Results_Format_Scans

' Sheets("Peptide Summary").Range("B:B,F:F,I:L,N:R").Delete Shift:=xlToLeft

C_cut = 0

'Move Sample Tally back to the front of columns

For NumToOpen_loop = 1 To NumToOpen

Columns(C_total + NumToOpen_loop - C_cut).Select

Selection.Cut

Columns(NumToOpen_loop).Select

Selection.Insert Shift:=xlToRight

Next NumToOpen_loop

Call Results_Format_Col(NumToOpen)

Range(Cells(1, 1), Cells(1, NumToOpen + 1).End(xlDown).Offset(0, -1)).Select

Selection.NumberFormat = "0"

' Delete the scan hits associated with Reverse Database sequences

delete_check = True

Do

If StrComp(Worksheets("Peptide Summary").Cells(2, C_gi + NumToOpen).Value, "-1", 1) = 0 Then

Worksheets("Peptide Summary").Rows(2).Delete

Else

delete_check = False

End If

Loop Until delete_check = False

' Tally occurances across the different samples and totals for each sample

Ri = Range(Cells(1, 1), Cells(1, NumToOpen + 1).End(xlDown).Offset(1, 0)).Rows.Count

Range(Cells(Ri, 1), Cells(Ri, 1)).FormulaR1C1 = "=COUNTIF(R[-" & Ri - 2 & "]C:R[-1]C,""<>0"")"

Range(Cells(Ri, 1), Cells(Ri, NumToOpen)).FillRight

Cells(Ri, NumToOpen + 1).FormulaR1C1 = "# Unique Peptides"

Range(Cells(2, NumToOpen + C_total - C_cut + 1), Cells(2, NumToOpen + C_total - C_cut + 1)).FormulaR1C1 = "=COUNTIF(RC[-" & (NumToOpen + C_total - C_cut) & "]:RC[-" & (C_total - C_cut + 1) & "],""<>0"")"

Range(Cells(2, NumToOpen + C_total - C_cut + 1), Cells(Ri - 1, NumToOpen + C_total - C_cut + 1)).FillDown

' Freeze the top header row

ActiveWindow.SplitRow = 1

ActiveWindow.FreezePanes = True

Range("A1").Select

End Sub

' Formats the “Protein Summary” sheet

Private Sub Results_Format_Protein(NumToOpen, C_total, C_gi)

Dim C_cut As Integer

C_cut = 8

Sheets("Protein Summary").Select

'Move Sample Tally to end of columns

Range(Cells(1, 1), Cells(1, NumToOpen + 1).End(xlDown).Offset(0, -1)).Select

Selection.Cut

Columns(NumToOpen + C_total + 1).Select

Selection.Insert Shift:=xlToRight

Sheets("Protein Summary").Range("B:I").Delete Shift:=xlToLeft

Range("A:B").NumberFormat = "0"

Range("C:C").NumberFormat = "0.00"

Range("D:D").NumberFormat = "@"

Columns("A:A").ColumnWidth = 9

Columns("B:C").ColumnWidth = 8

Columns("D:D").ColumnWidth = 100

' Sort by gi

Cells.Select

Selection.Sort Key1:=Range("A2"), Order1:=xlAscending, _

Header:=xlYes, OrderCustom:=1, MatchCase:=False, Orientation:=xlTopToBottom, DataOption1:=xlSortNormal, DataOption2:=xlSortNormal

' Remove Reverse hit result

If (Cells(2, NumToOpen + C_gi).Value = 0) Then

Rows("2:2").Delete Shift:=xlUp

End If

'Count number of total scans

Rlast = Range(Cells(1, 1), Cells(1, 1).End(xlDown)).Rows.Count

'Move Sample Tally back to the front of columns

For NumToOpen_loop = 1 To NumToOpen

Columns(C_total + NumToOpen_loop - C_cut).Select

Selection.Cut

Columns(NumToOpen_loop).Select

Selection.Insert Shift:=xlToRight

Next NumToOpen_loop

Call Results_Format_Col(NumToOpen)

' Tally occurances across the different samples

' Sort by Number of hits for first sample

ActiveWorkbook.Worksheets("Protein Summary").Sort.SortFields.Clear

ActiveWorkbook.Worksheets("Protein Summary").Sort.SortFields.Add Key:=Range("A2"), _

SortOn:=xlSortOnValues, Order:=xlDescending, DataOption:=xlSortNormal

With ActiveWorkbook.Worksheets("Protein Summary").Sort

.SetRange Range(Cells(1, 1), Cells(Rlast, C_total + NumToOpen - C_cut))

.Header = xlYes

.MatchCase = False

.Orientation = xlTopToBottom

.SortMethod = xlPinYin

.Apply

End With

Dim R_x As Integer

R_x = 1

For Count_loop = 2 To Rlast

If (Cells(Count_loop, 1).Value = "X") Then

R_x = R_x + 1

Else

Count_loop = Rlast

End If

Next Count_loop

If R_x > 1 Then

Range(Cells(2, 1), Cells(R_x, C_total + NumToOpen - C_cut)).Cut

Range(Cells(Rlast + 1, 1), Cells(Rlast + 1, C_total + NumToOpen - C_cut)).Insert Shift:=xlDown

End If

' Tally occurances across the different samples and totals for each sample

Ri = Range(Cells(1, 1), Cells(1, NumToOpen + 1).End(xlDown).Offset(1, 0)).Rows.Count

Range(Cells(Ri, 1), Cells(Ri, 1)).FormulaR1C1 = "=COUNTIF(R[-" & Ri - 2 & "]C:R[-1]C,"">0"")"

Range(Cells(Ri, 1), Cells(Ri, NumToOpen)).FillRight

Cells(Ri, NumToOpen + 1).FormulaR1C1 = "# Unique Proteins"

Range(Cells(2, NumToOpen + C_total - C_cut + 1), Cells(2, NumToOpen + C_total - C_cut + 1)).FormulaR1C1 = "=COUNTIF(RC[-" & (NumToOpen + C_total - C_cut) & "]:RC[-" & (C_total - C_cut + 1) & "],"">0"")"

Range(Cells(2, NumToOpen + C_total - C_cut + 1), Cells(Ri - 1, NumToOpen + C_total - C_cut + 1)).FillDown

' Freeze the top header row

ActiveWindow.SplitRow = 1

ActiveWindow.FreezePanes = True

Range("A1").Select

End Sub

' General formatting for the scan info

Private Sub Results_Format_Scans()

Range("A:B,F:G,J:J").Select

Selection.NumberFormat = "0"

Range("E:E,K:K").Select

Selection.NumberFormat = "0.00"

Range("D:D,H:I,L:L").Select

Selection.NumberFormat = "@"

Range("C:C").Select

Selection.NumberFormat = "0.00E+000"

Range("D:D,H:H,L:L").HorizontalAlignment = xlLeft

Range("A:C,E:E,J:K").HorizontalAlignment = xlRight

Range("F:G,I:I").HorizontalAlignment = xlCenter

Columns("A:A").ColumnWidth = 10

Columns("B:B").ColumnWidth = 8

Columns("C:C").ColumnWidth = 10

Columns("D:D").ColumnWidth = 26

Columns("E:E").ColumnWidth = 9

Columns("F:F").ColumnWidth = 8

Columns("G:G").ColumnWidth = 8

Columns("H:H").ColumnWidth = 26

Columns("I:I").ColumnWidth = 8

Columns("J:J").ColumnWidth = 8

Columns("K:K").ColumnWidth = 9

Columns("L:L").ColumnWidth = 60

Range("A1:L1").Select

Selection.NumberFormat = "@"

With Selection

.HorizontalAlignment = xlCenter

.VerticalAlignment = xlCenter

.WrapText = True

.Orientation = 0

.AddIndent = False

.IndentLevel = 0

.ShrinkToFit = False

.ReadingOrder = xlContext

.MergeCells = False

End With

With Selection.Font

.Name = "Arial"

.FontStyle = "Bold"

.Size = 10

.Strikethrough = False

.Superscript = False

.Subscript = False

.OutlineFont = False

.Shadow = False

.Underline = xlUnderlineStyleNone

.ColorIndex = xlAutomatic

End With

Selection.Borders(xlDiagonalDown).LineStyle = xlNone

Selection.Borders(xlDiagonalUp).LineStyle = xlNone

With Selection.Borders(xlEdgeLeft)

.LineStyle = xlContinuous

.Weight = xlMedium

.ColorIndex = xlAutomatic

End With

With Selection.Borders(xlEdgeTop)

.LineStyle = xlContinuous

.Weight = xlMedium

.ColorIndex = xlAutomatic

End With

With Selection.Borders(xlEdgeBottom)

.LineStyle = xlContinuous

.Weight = xlMedium

.ColorIndex = xlAutomatic

End With

With Selection.Borders(xlEdgeRight)

.LineStyle = xlContinuous

.Weight = xlMedium

.ColorIndex = xlAutomatic

End With

With Selection.Borders(xlInsideVertical)

.LineStyle = xlContinuous

.Weight = xlMedium

.ColorIndex = xlAutomatic

End With

With Selection.Interior

.ColorIndex = 39

.Pattern = xlSolid

.PatternColorIndex = xlAutomatic

End With

End Sub

' General formatting for the columns that keep track of from which datafile the scan/peptide/protein was identified

Private Sub Results_Format_Col(NumToOpen)

Range(Cells(1, 1), Cells(1, NumToOpen + 1).End(xlDown).Offset(0, -1)).Select

Selection.NumberFormat = "0.000"

Selection.Font.Bold = False

With Selection

.HorizontalAlignment = xlCenter

.AddIndent = False

.ShrinkToFit = False

.MergeCells = False

.ColumnWidth = 8

End With

Range(Cells(1, 1), Cells(1, NumToOpen)).Select

Selection.NumberFormat = "@"

With Selection

.HorizontalAlignment = xlCenter

.VerticalAlignment = xlCenter

.WrapText = True

.ShrinkToFit = False

.MergeCells = False

End With

With Selection.Font

.Name = "Arial"

.FontStyle = "Bold"

.Size = 10

End With

With Selection.Interior

.ColorIndex = 6

.Pattern = xlSolid

.PatternColorIndex = xlAutomatic

End With

With Selection.Borders(xlEdgeLeft)

.LineStyle = xlContinuous

.Weight = xlMedium

.ColorIndex = xlAutomatic

End With

With Selection.Borders(xlEdgeTop)

.LineStyle = xlContinuous

.Weight = xlMedium

.ColorIndex = xlAutomatic

End With

With Selection.Borders(xlEdgeBottom)

.LineStyle = xlContinuous

.Weight = xlMedium

.ColorIndex = xlAutomatic

End With

With Selection.Borders(xlEdgeRight)

.LineStyle = xlContinuous

.Weight = xlMedium

.ColorIndex = xlAutomatic

End With

With Selection.Borders(xlInsideVertical)

.LineStyle = xlContinuous

.Weight = xlMedium

.ColorIndex = xlAutomatic

End With

Range("A1").Select

End Sub
