# Supplementary material for: Proteomic Profile of Reversible Protein Oxidation Using PROP, Purification of Reversibly Oxidized Proteins
Source: PLoS One. 2012 Feb 28;7(2):e32527. doi: 10.1371/journal.pone.0032527 (PMC3289665; doi:10.1371/journal.pone.0032527)
Supplement: Analysis S1 — WebGestalt Results. This document contains the gene ontology (GO), KEGG enrichment and Wikipathways gene enrichment analysis obtained through using the web-based gene set analysis package WebGestalt [26]. (DOC) [file pone.0032527.s003.doc]

# GO Analysis


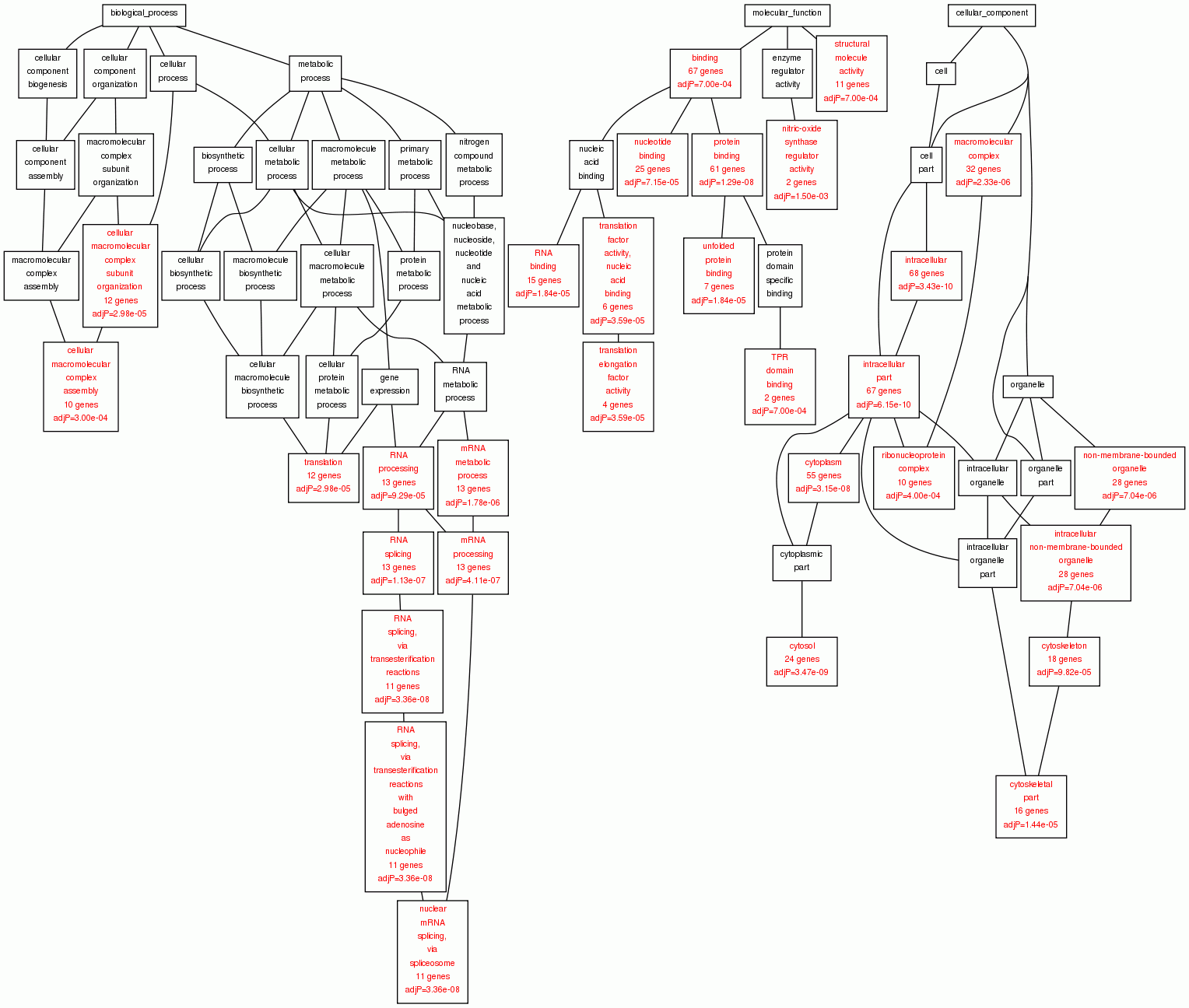


User file and parameters: User file: PROP_h2o2_entrezgene.txt, Organism: hsapiens, Id Type: entrezgene, Ref Set: entrezgene, Significance Level: Top10, Statistics Test: Hypergeometric, MTC: BH, Minimum: 2

The results for each enriched GO category are listed in this table. For each GO category, the first row lists its sub-root

(biological process, molecular function, or cellular component), category name, and corresponding GO ID. The second row lists

number of reference genes in the category (C), number of genes in the gene set and also in the category (O), expected number in the

category (E), Ratio of enrichment (R), p value from hypergeometric test (rawP), and p value adjusted by the multiple test adjustment (adjP).

Finally, genes in the category are listed. For each gene, the table lists the user uploaded ID and value (optional), Entrez ID,

Ensembl Gene Stable ID, Gene symbol, and description.

biological process RNA splicing, via transesterification reactions GO:0000375

C=160;O=11;E=0.77;R=14.32;rawP=2.41e-10;adjP=3.36e-08

6432 NA 6432 ENSG00000115875 SFRS7 splicing factor, arginine/serine-rich 7, 35kDa

4869 NA 4869 ENSG00000181163 NPM1 nucleophosmin (nucleolar phosphoprotein B23, numatrin)

6434 NA 6434 ENSG00000136527 TRA2B transformer 2 beta homolog (Drosophila)

3191 NA 3191 ENSG00000104824 HNRNPL heterogeneous nuclear ribonucleoprotein L

6428 NA 6428 ENSG00000112081 SFRS3 splicing factor, arginine/serine-rich 3

5093 NA 5093 ENSG00000169564 PCBP1 poly(rC) binding protein 1

220988 NA 220988 ENSG00000170144 HNRNPA3 heterogeneous nuclear ribonucleoprotein A3

8683 NA 8683 ENSG00000111786 SFRS9 splicing factor, arginine/serine-rich 9

5094 NA 5094 ENSG00000197111 PCBP2 poly(rC) binding protein 2

1207 NA 1207 ENSG00000074201 CLNS1A chloride channel, nucleotide-sensitive, 1A

6426 NA 6426 ENSG00000136450 SFRS1 splicing factor, arginine/serine-rich 1

biological process nuclear mRNA splicing, via spliceosome GO:0000398

C=160;O=11;E=0.77;R=14.32;rawP=2.41e-10;adjP=3.36e-08

6432 NA 6432 ENSG00000115875 SFRS7 splicing factor, arginine/serine-rich 7, 35kDa

4869 NA 4869 ENSG00000181163 NPM1 nucleophosmin (nucleolar phosphoprotein B23, numatrin)

6434 NA 6434 ENSG00000136527 TRA2B transformer 2 beta homolog (Drosophila)

3191 NA 3191 ENSG00000104824 HNRNPL heterogeneous nuclear ribonucleoprotein L

6428 NA 6428 ENSG00000112081 SFRS3 splicing factor, arginine/serine-rich 3

5093 NA 5093 ENSG00000169564 PCBP1 poly(rC) binding protein 1

220988 NA 220988 ENSG00000170144 HNRNPA3 heterogeneous nuclear ribonucleoprotein A3

8683 NA 8683 ENSG00000111786 SFRS9 splicing factor, arginine/serine-rich 9

5094 NA 5094 ENSG00000197111 PCBP2 poly(rC) binding protein 2

1207 NA 1207 ENSG00000074201 CLNS1A chloride channel, nucleotide-sensitive, 1A

6426 NA 6426 ENSG00000136450 SFRS1 splicing factor, arginine/serine-rich 1

biological process RNA splicing, via transesterification reactions with bulged adenosine as nucleophile GO:0000377

C=160;O=11;E=0.77;R=14.32;rawP=2.41e-10;adjP=3.36e-08

6432 NA 6432 ENSG00000115875 SFRS7 splicing factor, arginine/serine-rich 7, 35kDa

4869 NA 4869 ENSG00000181163 NPM1 nucleophosmin (nucleolar phosphoprotein B23, numatrin)

6434 NA 6434 ENSG00000136527 TRA2B transformer 2 beta homolog (Drosophila)

3191 NA 3191 ENSG00000104824 HNRNPL heterogeneous nuclear ribonucleoprotein L

6428 NA 6428 ENSG00000112081 SFRS3 splicing factor, arginine/serine-rich 3

5093 NA 5093 ENSG00000169564 PCBP1 poly(rC) binding protein 1

220988 NA 220988 ENSG00000170144 HNRNPA3 heterogeneous nuclear ribonucleoprotein A3

8683 NA 8683 ENSG00000111786 SFRS9 splicing factor, arginine/serine-rich 9

5094 NA 5094 ENSG00000197111 PCBP2 poly(rC) binding protein 2

1207 NA 1207 ENSG00000074201 CLNS1A chloride channel, nucleotide-sensitive, 1A

6426 NA 6426 ENSG00000136450 SFRS1 splicing factor, arginine/serine-rich 1

biological process RNA splicing GO:0008380

C=292;O=13;E=1.40;R=9.27;rawP=1.08e-09;adjP=1.13e-07

4841 NA 4841 ENSG00000147140 NONO non-POU domain containing, octamer-binding

6432 NA 6432 ENSG00000115875 SFRS7 splicing factor, arginine/serine-rich 7, 35kDa

8570 NA 8570 ENSG00000088247 KHSRP KH-type splicing regulatory protein

4869 NA 4869 ENSG00000181163 NPM1 nucleophosmin (nucleolar phosphoprotein B23, numatrin)

6434 NA 6434 ENSG00000136527 TRA2B transformer 2 beta homolog (Drosophila)

3191 NA 3191 ENSG00000104824 HNRNPL heterogeneous nuclear ribonucleoprotein L

6428 NA 6428 ENSG00000112081 SFRS3 splicing factor, arginine/serine-rich 3

5093 NA 5093 ENSG00000169564 PCBP1 poly(rC) binding protein 1

220988 NA 220988 ENSG00000170144 HNRNPA3 heterogeneous nuclear ribonucleoprotein A3

8683 NA 8683 ENSG00000111786 SFRS9 splicing factor, arginine/serine-rich 9

5094 NA 5094 ENSG00000197111 PCBP2 poly(rC) binding protein 2

1207 NA 1207 ENSG00000074201 CLNS1A chloride channel, nucleotide-sensitive, 1A

6426 NA 6426 ENSG00000136450 SFRS1 splicing factor, arginine/serine-rich 1

biological process mRNA processing GO:0006397

C=331;O=13;E=1.59;R=8.18;rawP=4.92e-09;adjP=4.11e-07

4841 NA 4841 ENSG00000147140 NONO non-POU domain containing, octamer-binding

6432 NA 6432 ENSG00000115875 SFRS7 splicing factor, arginine/serine-rich 7, 35kDa

8570 NA 8570 ENSG00000088247 KHSRP KH-type splicing regulatory protein

4869 NA 4869 ENSG00000181163 NPM1 nucleophosmin (nucleolar phosphoprotein B23, numatrin)

6434 NA 6434 ENSG00000136527 TRA2B transformer 2 beta homolog (Drosophila)

3191 NA 3191 ENSG00000104824 HNRNPL heterogeneous nuclear ribonucleoprotein L

6428 NA 6428 ENSG00000112081 SFRS3 splicing factor, arginine/serine-rich 3

5093 NA 5093 ENSG00000169564 PCBP1 poly(rC) binding protein 1

220988 NA 220988 ENSG00000170144 HNRNPA3 heterogeneous nuclear ribonucleoprotein A3

8683 NA 8683 ENSG00000111786 SFRS9 splicing factor, arginine/serine-rich 9

5094 NA 5094 ENSG00000197111 PCBP2 poly(rC) binding protein 2

1207 NA 1207 ENSG00000074201 CLNS1A chloride channel, nucleotide-sensitive, 1A

6426 NA 6426 ENSG00000136450 SFRS1 splicing factor, arginine/serine-rich 1

biological process mRNA metabolic process GO:0016071

C=380;O=13;E=1.82;R=7.12;rawP=2.55e-08;adjP=1.78e-06

4841 NA 4841 ENSG00000147140 NONO non-POU domain containing, octamer-binding

6432 NA 6432 ENSG00000115875 SFRS7 splicing factor, arginine/serine-rich 7, 35kDa

8570 NA 8570 ENSG00000088247 KHSRP KH-type splicing regulatory protein

4869 NA 4869 ENSG00000181163 NPM1 nucleophosmin (nucleolar phosphoprotein B23, numatrin)

6434 NA 6434 ENSG00000136527 TRA2B transformer 2 beta homolog (Drosophila)

3191 NA 3191 ENSG00000104824 HNRNPL heterogeneous nuclear ribonucleoprotein L

6428 NA 6428 ENSG00000112081 SFRS3 splicing factor, arginine/serine-rich 3

5093 NA 5093 ENSG00000169564 PCBP1 poly(rC) binding protein 1

220988 NA 220988 ENSG00000170144 HNRNPA3 heterogeneous nuclear ribonucleoprotein A3

8683 NA 8683 ENSG00000111786 SFRS9 splicing factor, arginine/serine-rich 9

5094 NA 5094 ENSG00000197111 PCBP2 poly(rC) binding protein 2

1207 NA 1207 ENSG00000074201 CLNS1A chloride channel, nucleotide-sensitive, 1A

6426 NA 6426 ENSG00000136450 SFRS1 splicing factor, arginine/serine-rich 1

biological process cellular macromolecular complex subunit organization GO:0034621

C=415;O=12;E=1.99;R=6.02;rawP=5.71e-07;adjP=2.98e-05

1984 NA 1984 ENSG00000132507 EIF5A eukaryotic translation initiation factor 5A

3692 NA 3692 ENSG00000126005 EIF6 eukaryotic translation initiation factor 6

3146 NA 3146 ENSG00000189403 HMGB1 high-mobility group box 1

10383 NA 10383 ENSG00000188229 TUBB2C tubulin, beta 2C

4869 NA 4869 ENSG00000181163 NPM1 nucleophosmin (nucleolar phosphoprotein B23, numatrin)

3320 NA 3320 ENSG00000080824 HSP90AA1 heat shock protein 90kDa alpha (cytosolic), class A member 1

832 NA 832 ENSG00000077549 CAPZB capping protein (actin filament) muscle Z-line, beta

8683 NA 8683 ENSG00000111786 SFRS9 splicing factor, arginine/serine-rich 9

1072 NA 1072 ENSG00000172757 CFL1 cofilin 1 (non-muscle)

1207 NA 1207 ENSG00000074201 CLNS1A chloride channel, nucleotide-sensitive, 1A

203068 NA 203068 ENSG00000196230 TUBB tubulin, beta

6426 NA 6426 ENSG00000136450 SFRS1 splicing factor, arginine/serine-rich 1

biological process translation GO:0006412

C=410;O=12;E=1.97;R=6.10;rawP=5.02e-07;adjP=2.98e-05

1984 NA 1984 ENSG00000132507 EIF5A eukaryotic translation initiation factor 5A

3692 NA 3692 ENSG00000126005 EIF6 eukaryotic translation initiation factor 6

2617 NA 2617 ENSG00000106105 GARS glycyl-tRNA synthetase

1975 NA 1975 ENSG00000063046 EIF4B eukaryotic translation initiation factor 4B

1938 NA 1938 ENSG00000167658 EEF2 eukaryotic translation elongation factor 2

23708 NA 23708 ENSG00000189369 GSPT2 G1 to S phase transition 2

6176 NA 6176 ENSG00000137818 RPLP1 ribosomal protein, large, P1

6206 NA 6206 ENSG00000112306 RPS12 ribosomal protein S12

1937 NA 1937 ENSG00000149016 EEF1G eukaryotic translation elongation factor 1 gamma

3315 NA 3315 ENSG00000106211 HSPB1 heat shock 27kDa protein 1

136319 NA 136319 ENSG00000105887 MTPN myotrophin

1933 NA 1933 ENSG00000114942 EEF1B2 eukaryotic translation elongation factor 1 beta 2

biological process RNA processing GO:0006396

C=556;O=13;E=2.67;R=4.87;rawP=2.00e-06;adjP=9.29e-05

4841 NA 4841 ENSG00000147140 NONO non-POU domain containing, octamer-binding

6432 NA 6432 ENSG00000115875 SFRS7 splicing factor, arginine/serine-rich 7, 35kDa

8570 NA 8570 ENSG00000088247 KHSRP KH-type splicing regulatory protein

4869 NA 4869 ENSG00000181163 NPM1 nucleophosmin (nucleolar phosphoprotein B23, numatrin)

6434 NA 6434 ENSG00000136527 TRA2B transformer 2 beta homolog (Drosophila)

3191 NA 3191 ENSG00000104824 HNRNPL heterogeneous nuclear ribonucleoprotein L

6428 NA 6428 ENSG00000112081 SFRS3 splicing factor, arginine/serine-rich 3

5093 NA 5093 ENSG00000169564 PCBP1 poly(rC) binding protein 1

220988 NA 220988 ENSG00000170144 HNRNPA3 heterogeneous nuclear ribonucleoprotein A3

8683 NA 8683 ENSG00000111786 SFRS9 splicing factor, arginine/serine-rich 9

5094 NA 5094 ENSG00000197111 PCBP2 poly(rC) binding protein 2

1207 NA 1207 ENSG00000074201 CLNS1A chloride channel, nucleotide-sensitive, 1A

6426 NA 6426 ENSG00000136450 SFRS1 splicing factor, arginine/serine-rich 1

biological process cellular macromolecular complex assembly GO:0034622

C=354;O=10;E=1.70;R=5.88;rawP=6.77e-06;adjP=0.0003

3692 NA 3692 ENSG00000126005 EIF6 eukaryotic translation initiation factor 6

3146 NA 3146 ENSG00000189403 HMGB1 high-mobility group box 1

10383 NA 10383 ENSG00000188229 TUBB2C tubulin, beta 2C

4869 NA 4869 ENSG00000181163 NPM1 nucleophosmin (nucleolar phosphoprotein B23, numatrin)

3320 NA 3320 ENSG00000080824 HSP90AA1 heat shock protein 90kDa alpha (cytosolic), class A member 1

832 NA 832 ENSG00000077549 CAPZB capping protein (actin filament) muscle Z-line, beta

1207 NA 1207 ENSG00000074201 CLNS1A chloride channel, nucleotide-sensitive, 1A

203068 NA 203068 ENSG00000196230 TUBB tubulin, beta

8683 NA 8683 ENSG00000111786 SFRS9 splicing factor, arginine/serine-rich 9

6426 NA 6426 ENSG00000136450 SFRS1 splicing factor, arginine/serine-rich 1

molecular function protein binding GO:0005515

C=8041;O=61;E=36.11;R=1.69;rawP=1.68e-10;adjP=1.29e-08

3146 NA 3146 ENSG00000189403 HMGB1 high-mobility group box 1

7295 NA 7295 ENSG00000136810 TXN thioredoxin

3068 NA 3068 ENSG00000143321 HDGF hepatoma-derived growth factor (high-mobility group protein 1-like)

1674 NA 1674 ENSG00000175084 DES desmin

10383 NA 10383 ENSG00000188229 TUBB2C tubulin, beta 2C

23708 NA 23708 ENSG00000189369 GSPT2 G1 to S phase transition 2

7086 NA 7086 ENSG00000163931 TKT transketolase

4869 NA 4869 ENSG00000181163 NPM1 nucleophosmin (nucleolar phosphoprotein B23, numatrin)

5715 NA 5715 ENSG00000110801 PSMD9 proteasome (prosome, macropain) 26S subunit, non-ATPase, 9

5093 NA 5093 ENSG00000169564 PCBP1 poly(rC) binding protein 1

1937 NA 1937 ENSG00000149016 EEF1G eukaryotic translation elongation factor 1 gamma

140465 NA 140465 ENSG00000196465 MYL6B myosin, light chain 6B, alkali, smooth muscle and non-muscle

220988 NA 220988 ENSG00000170144 HNRNPA3 heterogeneous nuclear ribonucleoprotein A3

11315 NA 11315 ENSG00000116288 PARK7 Parkinson disease (autosomal recessive, early onset) 7

4000 NA 4000 ENSG00000160789 LMNA lamin A/C

5478 NA 5478 ENSG00000196262 PPIA peptidylprolyl isomerase A (cyclophilin A)

3868 NA 3868 ENSG00000186832 KRT16 keratin 16

1933 NA 1933 ENSG00000114942 EEF1B2 eukaryotic translation elongation factor 1 beta 2

3692 NA 3692 ENSG00000126005 EIF6 eukaryotic translation initiation factor 6

1938 NA 1938 ENSG00000167658 EEF2 eukaryotic translation elongation factor 2

10606 NA 10606 ENSG00000128050 PAICS phosphoribosylaminoimidazole carboxylase, phosphoribosylaminoimidazole succinocarboxamide synthetase

1854 NA 1854 ENSG00000128951 DUT deoxyuridine triphosphatase

6432 NA 6432 ENSG00000115875 SFRS7 splicing factor, arginine/serine-rich 7, 35kDa

4831 NA 4831 ENSG00000011052 NME2 non-metastatic cells 2, protein (NM23B) expressed in

6434 NA 6434 ENSG00000136527 TRA2B transformer 2 beta homolog (Drosophila)

3191 NA 3191 ENSG00000104824 HNRNPL heterogeneous nuclear ribonucleoprotein L

8407 NA 8407 ENSG00000158710 TAGLN2 transgelin 2

10728 NA 10728 ENSG00000110958 PTGES3 prostaglandin E synthase 3 (cytosolic)

3320 NA 3320 ENSG00000080824 HSP90AA1 heat shock protein 90kDa alpha (cytosolic), class A member 1

11164 NA 11164 ENSG00000165609 NUDT5 nudix (nucleoside diphosphate linked moiety X)-type motif 5

136319 NA 136319 ENSG00000105887 MTPN myotrophin

1072 NA 1072 ENSG00000172757 CFL1 cofilin 1 (non-muscle)

5094 NA 5094 ENSG00000197111 PCBP2 poly(rC) binding protein 2

4634 NA 4634 ENSG00000160808 MYL3 myosin, light chain 3, alkali; ventricular, skeletal, slow

1984 NA 1984 ENSG00000132507 EIF5A eukaryotic translation initiation factor 5A

2617 NA 2617 ENSG00000106105 GARS glycyl-tRNA synthetase

4841 NA 4841 ENSG00000147140 NONO non-POU domain containing, octamer-binding

6275 NA 6275 ENSG00000196154 S100A4 S100 calcium binding protein A4

3326 NA 3326 ENSG00000096384 HSP90AB1 heat shock protein 90kDa alpha (cytosolic), class B member 1

475 NA 475 ENSG00000177556 ATOX1 ATX1 antioxidant protein 1 homolog (yeast)

1892 NA 1892 ENSG00000127884 ECHS1 enoyl Coenzyme A hydratase, short chain, 1, mitochondrial

7417 NA 7417 ENSG00000165637 VDAC2 voltage-dependent anion channel 2

3956 NA 3956 ENSG00000100097 LGALS1 lectin, galactoside-binding, soluble, 1

10971 NA 10971 ENSG00000134308 YWHAQ tyrosine 3-monooxygenase/tryptophan 5-monooxygenase activation protein, theta polypeptide

7431 NA 7431 ENSG00000026025 VIM vimentin

5315 NA 5315 ENSG00000067225 PKM2 pyruvate kinase, muscle

11335 NA 11335 ENSG00000122565 CBX3 chromobox homolog 3 (HP1 gamma homolog, Drosophila)

7520 NA 7520 ENSG00000079246 XRCC5 X-ray repair complementing defective repair in Chinese hamster cells 5 (double-strand-break rejoining)

6426 NA 6426 ENSG00000136450 SFRS1 splicing factor, arginine/serine-rich 1

23589 NA 23589 ENSG00000153048 CARHSP1 calcium regulated heat stable protein 1, 24kDa

3313 NA 3313 ENSG00000113013 HSPA9 heat shock 70kDa protein 9 (mortalin)

4830 NA 4830 ENSG00000011052 NME1 non-metastatic cells 1, protein (NM23A) expressed in

6624 NA 6624 ENSG00000075618 FSCN1 fascin homolog 1, actin-bundling protein (Strongylocentrotus purpuratus)

6176 NA 6176 ENSG00000137818 RPLP1 ribosomal protein, large, P1

8570 NA 8570 ENSG00000088247 KHSRP KH-type splicing regulatory protein

52 NA 52 ENSG00000143727 ACP1 acid phosphatase 1, soluble

2745 NA 2745 ENSG00000173221 GLRX glutaredoxin (thioltransferase)

6428 NA 6428 ENSG00000112081 SFRS3 splicing factor, arginine/serine-rich 3

832 NA 832 ENSG00000077549 CAPZB capping protein (actin filament) muscle Z-line, beta

3315 NA 3315 ENSG00000106211 HSPB1 heat shock 27kDa protein 1

203068 NA 203068 ENSG00000196230 TUBB tubulin, beta

molecular function unfolded protein binding GO:0051082

C=113;O=7;E=0.51;R=13.79;rawP=7.15e-07;adjP=1.84e-05

4869 NA 4869 ENSG00000181163 NPM1 nucleophosmin (nucleolar phosphoprotein B23, numatrin)

3313 NA 3313 ENSG00000113013 HSPA9 heat shock 70kDa protein 9 (mortalin)

10728 NA 10728 ENSG00000110958 PTGES3 prostaglandin E synthase 3 (cytosolic)

3320 NA 3320 ENSG00000080824 HSP90AA1 heat shock protein 90kDa alpha (cytosolic), class A member 1

10383 NA 10383 ENSG00000188229 TUBB2C tubulin, beta 2C

3326 NA 3326 ENSG00000096384 HSP90AB1 heat shock protein 90kDa alpha (cytosolic), class B member 1

5478 NA 5478 ENSG00000196262 PPIA peptidylprolyl isomerase A (cyclophilin A)

molecular function RNA binding GO:0003723

C=715;O=15;E=3.21;R=4.67;rawP=4.88e-07;adjP=1.84e-05

1984 NA 1984 ENSG00000132507 EIF5A eukaryotic translation initiation factor 5A

4841 NA 4841 ENSG00000147140 NONO non-POU domain containing, octamer-binding

1975 NA 1975 ENSG00000063046 EIF4B eukaryotic translation initiation factor 4B

6432 NA 6432 ENSG00000115875 SFRS7 splicing factor, arginine/serine-rich 7, 35kDa

6176 NA 6176 ENSG00000137818 RPLP1 ribosomal protein, large, P1

8570 NA 8570 ENSG00000088247 KHSRP KH-type splicing regulatory protein

4869 NA 4869 ENSG00000181163 NPM1 nucleophosmin (nucleolar phosphoprotein B23, numatrin)

6434 NA 6434 ENSG00000136527 TRA2B transformer 2 beta homolog (Drosophila)

3191 NA 3191 ENSG00000104824 HNRNPL heterogeneous nuclear ribonucleoprotein L

6428 NA 6428 ENSG00000112081 SFRS3 splicing factor, arginine/serine-rich 3

5093 NA 5093 ENSG00000169564 PCBP1 poly(rC) binding protein 1

220988 NA 220988 ENSG00000170144 HNRNPA3 heterogeneous nuclear ribonucleoprotein A3

8683 NA 8683 ENSG00000111786 SFRS9 splicing factor, arginine/serine-rich 9

5094 NA 5094 ENSG00000197111 PCBP2 poly(rC) binding protein 2

6426 NA 6426 ENSG00000136450 SFRS1 splicing factor, arginine/serine-rich 1

molecular function translation factor activity, nucleic acid binding GO:0008135

C=86;O=6;E=0.39;R=15.53;rawP=2.33e-06;adjP=3.59e-05

1984 NA 1984 ENSG00000132507 EIF5A eukaryotic translation initiation factor 5A

3692 NA 3692 ENSG00000126005 EIF6 eukaryotic translation initiation factor 6

1975 NA 1975 ENSG00000063046 EIF4B eukaryotic translation initiation factor 4B

1938 NA 1938 ENSG00000167658 EEF2 eukaryotic translation elongation factor 2

1937 NA 1937 ENSG00000149016 EEF1G eukaryotic translation elongation factor 1 gamma

1933 NA 1933 ENSG00000114942 EEF1B2 eukaryotic translation elongation factor 1 beta 2

molecular function translation elongation factor activity GO:0003746

C=21;O=4;E=0.09;R=42.41;rawP=2.10e-06;adjP=3.59e-05

1984 NA 1984 ENSG00000132507 EIF5A eukaryotic translation initiation factor 5A

1938 NA 1938 ENSG00000167658 EEF2 eukaryotic translation elongation factor 2

1937 NA 1937 ENSG00000149016 EEF1G eukaryotic translation elongation factor 1 gamma

1933 NA 1933 ENSG00000114942 EEF1B2 eukaryotic translation elongation factor 1 beta 2

molecular function nucleotide binding GO:0000166

C=2219;O=25;E=9.97;R=2.51;rawP=5.57e-06;adjP=7.15e-05

4841 NA 4841 ENSG00000147140 NONO non-POU domain containing, octamer-binding

2617 NA 2617 ENSG00000106105 GARS glycyl-tRNA synthetase

1975 NA 1975 ENSG00000063046 EIF4B eukaryotic translation initiation factor 4B

3068 NA 3068 ENSG00000143321 HDGF hepatoma-derived growth factor (high-mobility group protein 1-like)

10383 NA 10383 ENSG00000188229 TUBB2C tubulin, beta 2C

3326 NA 3326 ENSG00000096384 HSP90AB1 heat shock protein 90kDa alpha (cytosolic), class B member 1

23708 NA 23708 ENSG00000189369 GSPT2 G1 to S phase transition 2

7417 NA 7417 ENSG00000165637 VDAC2 voltage-dependent anion channel 2

5315 NA 5315 ENSG00000067225 PKM2 pyruvate kinase, muscle

26227 NA 26227 ENSG00000092621 PHGDH phosphoglycerate dehydrogenase

220988 NA 220988 ENSG00000170144 HNRNPA3 heterogeneous nuclear ribonucleoprotein A3

7520 NA 7520 ENSG00000079246 XRCC5 X-ray repair complementing defective repair in Chinese hamster cells 5 (double-strand-break rejoining)

6426 NA 6426 ENSG00000136450 SFRS1 splicing factor, arginine/serine-rich 1

3313 NA 3313 ENSG00000113013 HSPA9 heat shock 70kDa protein 9 (mortalin)

4830 NA 4830 ENSG00000011052 NME1 non-metastatic cells 1, protein (NM23A) expressed in

1938 NA 1938 ENSG00000167658 EEF2 eukaryotic translation elongation factor 2

10606 NA 10606 ENSG00000128050 PAICS phosphoribosylaminoimidazole carboxylase, phosphoribosylaminoimidazole succinocarboxamide synthetase

6432 NA 6432 ENSG00000115875 SFRS7 splicing factor, arginine/serine-rich 7, 35kDa

4831 NA 4831 ENSG00000011052 NME2 non-metastatic cells 2, protein (NM23B) expressed in

6434 NA 6434 ENSG00000136527 TRA2B transformer 2 beta homolog (Drosophila)

3191 NA 3191 ENSG00000104824 HNRNPL heterogeneous nuclear ribonucleoprotein L

6428 NA 6428 ENSG00000112081 SFRS3 splicing factor, arginine/serine-rich 3

3320 NA 3320 ENSG00000080824 HSP90AA1 heat shock protein 90kDa alpha (cytosolic), class A member 1

8683 NA 8683 ENSG00000111786 SFRS9 splicing factor, arginine/serine-rich 9

203068 NA 203068 ENSG00000196230 TUBB tubulin, beta

molecular function structural molecule activity GO:0005198

C=603;O=11;E=2.71;R=4.06;rawP=7.15e-05;adjP=0.0007

1674 NA 1674 ENSG00000175084 DES desmin

10383 NA 10383 ENSG00000188229 TUBB2C tubulin, beta 2C

6176 NA 6176 ENSG00000137818 RPLP1 ribosomal protein, large, P1

4637 NA 4637 ENSG00000092841 MYL6 myosin, light chain 6, alkali, smooth muscle and non-muscle

7431 NA 7431 ENSG00000026025 VIM vimentin

6206 NA 6206 ENSG00000112306 RPS12 ribosomal protein S12

140465 NA 140465 ENSG00000196465 MYL6B myosin, light chain 6B, alkali, smooth muscle and non-muscle

3868 NA 3868 ENSG00000186832 KRT16 keratin 16

203068 NA 203068 ENSG00000196230 TUBB tubulin, beta

4000 NA 4000 ENSG00000160789 LMNA lamin A/C

4634 NA 4634 ENSG00000160808 MYL3 myosin, light chain 3, alkali; ventricular, skeletal, slow

molecular function TPR domain binding GO:0030911

C=3;O=2;E=0.01;R=148.44;rawP=5.95e-05;adjP=0.0007

3320 NA 3320 ENSG00000080824 HSP90AA1 heat shock protein 90kDa alpha (cytosolic), class A member 1

3326 NA 3326 ENSG00000096384 HSP90AB1 heat shock protein 90kDa alpha (cytosolic), class B member 1

molecular function binding GO:0005488

C=12465;O=67;E=55.98;R=1.20;rawP=7.60e-05;adjP=0.0007

7295 NA 7295 ENSG00000136810 TXN thioredoxin

3068 NA 3068 ENSG00000143321 HDGF hepatoma-derived growth factor (high-mobility group protein 1-like)

1674 NA 1674 ENSG00000175084 DES desmin

10383 NA 10383 ENSG00000188229 TUBB2C tubulin, beta 2C

4869 NA 4869 ENSG00000181163 NPM1 nucleophosmin (nucleolar phosphoprotein B23, numatrin)

5093 NA 5093 ENSG00000169564 PCBP1 poly(rC) binding protein 1

1937 NA 1937 ENSG00000149016 EEF1G eukaryotic translation elongation factor 1 gamma

220988 NA 220988 ENSG00000170144 HNRNPA3 heterogeneous nuclear ribonucleoprotein A3

11315 NA 11315 ENSG00000116288 PARK7 Parkinson disease (autosomal recessive, early onset) 7

3868 NA 3868 ENSG00000186832 KRT16 keratin 16

1933 NA 1933 ENSG00000114942 EEF1B2 eukaryotic translation elongation factor 1 beta 2

79174 NA 79174 ENSG00000184164 CRELD2 cysteine-rich with EGF-like domains 2

1854 NA 1854 ENSG00000128951 DUT deoxyuridine triphosphatase

4831 NA 4831 ENSG00000011052 NME2 non-metastatic cells 2, protein (NM23B) expressed in

6434 NA 6434 ENSG00000136527 TRA2B transformer 2 beta homolog (Drosophila)

4637 NA 4637 ENSG00000092841 MYL6 myosin, light chain 6, alkali, smooth muscle and non-muscle

3191 NA 3191 ENSG00000104824 HNRNPL heterogeneous nuclear ribonucleoprotein L

136319 NA 136319 ENSG00000105887 MTPN myotrophin

1072 NA 1072 ENSG00000172757 CFL1 cofilin 1 (non-muscle)

5094 NA 5094 ENSG00000197111 PCBP2 poly(rC) binding protein 2

2617 NA 2617 ENSG00000106105 GARS glycyl-tRNA synthetase

4841 NA 4841 ENSG00000147140 NONO non-POU domain containing, octamer-binding

6275 NA 6275 ENSG00000196154 S100A4 S100 calcium binding protein A4

3326 NA 3326 ENSG00000096384 HSP90AB1 heat shock protein 90kDa alpha (cytosolic), class B member 1

475 NA 475 ENSG00000177556 ATOX1 ATX1 antioxidant protein 1 homolog (yeast)

7417 NA 7417 ENSG00000165637 VDAC2 voltage-dependent anion channel 2

3956 NA 3956 ENSG00000100097 LGALS1 lectin, galactoside-binding, soluble, 1

10971 NA 10971 ENSG00000134308 YWHAQ tyrosine 3-monooxygenase/tryptophan 5-monooxygenase activation protein, theta polypeptide

7431 NA 7431 ENSG00000026025 VIM vimentin

6426 NA 6426 ENSG00000136450 SFRS1 splicing factor, arginine/serine-rich 1

23589 NA 23589 ENSG00000153048 CARHSP1 calcium regulated heat stable protein 1, 24kDa

55250 NA 55250 ENSG00000134759 ELP2 elongation protein 2 homolog (S. cerevisiae)

4830 NA 4830 ENSG00000011052 NME1 non-metastatic cells 1, protein (NM23A) expressed in

6176 NA 6176 ENSG00000137818 RPLP1 ribosomal protein, large, P1

52 NA 52 ENSG00000143727 ACP1 acid phosphatase 1, soluble

3315 NA 3315 ENSG00000106211 HSPB1 heat shock 27kDa protein 1

203068 NA 203068 ENSG00000196230 TUBB tubulin, beta

3146 NA 3146 ENSG00000189403 HMGB1 high-mobility group box 1

23708 NA 23708 ENSG00000189369 GSPT2 G1 to S phase transition 2

7086 NA 7086 ENSG00000163931 TKT transketolase

26227 NA 26227 ENSG00000092621 PHGDH phosphoglycerate dehydrogenase

5715 NA 5715 ENSG00000110801 PSMD9 proteasome (prosome, macropain) 26S subunit, non-ATPase, 9

140465 NA 140465 ENSG00000196465 MYL6B myosin, light chain 6B, alkali, smooth muscle and non-muscle

5478 NA 5478 ENSG00000196262 PPIA peptidylprolyl isomerase A (cyclophilin A)

4000 NA 4000 ENSG00000160789 LMNA lamin A/C

3692 NA 3692 ENSG00000126005 EIF6 eukaryotic translation initiation factor 6

1938 NA 1938 ENSG00000167658 EEF2 eukaryotic translation elongation factor 2

10606 NA 10606 ENSG00000128050 PAICS phosphoribosylaminoimidazole carboxylase, phosphoribosylaminoimidazole succinocarboxamide synthetase

6432 NA 6432 ENSG00000115875 SFRS7 splicing factor, arginine/serine-rich 7, 35kDa

11164 NA 11164 ENSG00000165609 NUDT5 nudix (nucleoside diphosphate linked moiety X)-type motif 5

3320 NA 3320 ENSG00000080824 HSP90AA1 heat shock protein 90kDa alpha (cytosolic), class A member 1

10728 NA 10728 ENSG00000110958 PTGES3 prostaglandin E synthase 3 (cytosolic)

8407 NA 8407 ENSG00000158710 TAGLN2 transgelin 2

4634 NA 4634 ENSG00000160808 MYL3 myosin, light chain 3, alkali; ventricular, skeletal, slow

1984 NA 1984 ENSG00000132507 EIF5A eukaryotic translation initiation factor 5A

1975 NA 1975 ENSG00000063046 EIF4B eukaryotic translation initiation factor 4B

1892 NA 1892 ENSG00000127884 ECHS1 enoyl Coenzyme A hydratase, short chain, 1, mitochondrial

5315 NA 5315 ENSG00000067225 PKM2 pyruvate kinase, muscle

7520 NA 7520 ENSG00000079246 XRCC5 X-ray repair complementing defective repair in Chinese hamster cells 5 (double-strand-break rejoining)

11335 NA 11335 ENSG00000122565 CBX3 chromobox homolog 3 (HP1 gamma homolog, Drosophila)

3313 NA 3313 ENSG00000113013 HSPA9 heat shock 70kDa protein 9 (mortalin)

6624 NA 6624 ENSG00000075618 FSCN1 fascin homolog 1, actin-bundling protein (Strongylocentrotus purpuratus)

8570 NA 8570 ENSG00000088247 KHSRP KH-type splicing regulatory protein

2745 NA 2745 ENSG00000173221 GLRX glutaredoxin (thioltransferase)

6428 NA 6428 ENSG00000112081 SFRS3 splicing factor, arginine/serine-rich 3

832 NA 832 ENSG00000077549 CAPZB capping protein (actin filament) muscle Z-line, beta

8683 NA 8683 ENSG00000111786 SFRS9 splicing factor, arginine/serine-rich 9

molecular function nitric-oxide synthase regulator activity GO:0030235

C=5;O=2;E=0.02;R=89.07;rawP=0.0002;adjP=0.0015

3320 NA 3320 ENSG00000080824 HSP90AA1 heat shock protein 90kDa alpha (cytosolic), class A member 1

3326 NA 3326 ENSG00000096384 HSP90AB1 heat shock protein 90kDa alpha (cytosolic), class B member 1

cellular component intracellular GO:0005622

C=10940;O=68;E=46.13;R=1.47;rawP=3.24e-12;adjP=3.43e-10

7295 NA 7295 ENSG00000136810 TXN thioredoxin

3068 NA 3068 ENSG00000143321 HDGF hepatoma-derived growth factor (high-mobility group protein 1-like)

1674 NA 1674 ENSG00000175084 DES desmin

10383 NA 10383 ENSG00000188229 TUBB2C tubulin, beta 2C

4869 NA 4869 ENSG00000181163 NPM1 nucleophosmin (nucleolar phosphoprotein B23, numatrin)

5093 NA 5093 ENSG00000169564 PCBP1 poly(rC) binding protein 1

1937 NA 1937 ENSG00000149016 EEF1G eukaryotic translation elongation factor 1 gamma

220988 NA 220988 ENSG00000170144 HNRNPA3 heterogeneous nuclear ribonucleoprotein A3

11315 NA 11315 ENSG00000116288 PARK7 Parkinson disease (autosomal recessive, early onset) 7

3868 NA 3868 ENSG00000186832 KRT16 keratin 16

1933 NA 1933 ENSG00000114942 EEF1B2 eukaryotic translation elongation factor 1 beta 2

79174 NA 79174 ENSG00000184164 CRELD2 cysteine-rich with EGF-like domains 2

1854 NA 1854 ENSG00000128951 DUT deoxyuridine triphosphatase

4831 NA 4831 ENSG00000011052 NME2 non-metastatic cells 2, protein (NM23B) expressed in

6434 NA 6434 ENSG00000136527 TRA2B transformer 2 beta homolog (Drosophila)

4637 NA 4637 ENSG00000092841 MYL6 myosin, light chain 6, alkali, smooth muscle and non-muscle

3191 NA 3191 ENSG00000104824 HNRNPL heterogeneous nuclear ribonucleoprotein L

136319 NA 136319 ENSG00000105887 MTPN myotrophin

1072 NA 1072 ENSG00000172757 CFL1 cofilin 1 (non-muscle)

5094 NA 5094 ENSG00000197111 PCBP2 poly(rC) binding protein 2

2617 NA 2617 ENSG00000106105 GARS glycyl-tRNA synthetase

4841 NA 4841 ENSG00000147140 NONO non-POU domain containing, octamer-binding

6275 NA 6275 ENSG00000196154 S100A4 S100 calcium binding protein A4

3326 NA 3326 ENSG00000096384 HSP90AB1 heat shock protein 90kDa alpha (cytosolic), class B member 1

475 NA 475 ENSG00000177556 ATOX1 ATX1 antioxidant protein 1 homolog (yeast)

7417 NA 7417 ENSG00000165637 VDAC2 voltage-dependent anion channel 2

3956 NA 3956 ENSG00000100097 LGALS1 lectin, galactoside-binding, soluble, 1

10971 NA 10971 ENSG00000134308 YWHAQ tyrosine 3-monooxygenase/tryptophan 5-monooxygenase activation protein, theta polypeptide

7431 NA 7431 ENSG00000026025 VIM vimentin

1207 NA 1207 ENSG00000074201 CLNS1A chloride channel, nucleotide-sensitive, 1A

6426 NA 6426 ENSG00000136450 SFRS1 splicing factor, arginine/serine-rich 1

23589 NA 23589 ENSG00000153048 CARHSP1 calcium regulated heat stable protein 1, 24kDa

4830 NA 4830 ENSG00000011052 NME1 non-metastatic cells 1, protein (NM23A) expressed in

55250 NA 55250 ENSG00000134759 ELP2 elongation protein 2 homolog (S. cerevisiae)

6176 NA 6176 ENSG00000137818 RPLP1 ribosomal protein, large, P1

52 NA 52 ENSG00000143727 ACP1 acid phosphatase 1, soluble

3315 NA 3315 ENSG00000106211 HSPB1 heat shock 27kDa protein 1

203068 NA 203068 ENSG00000196230 TUBB tubulin, beta

3146 NA 3146 ENSG00000189403 HMGB1 high-mobility group box 1

23708 NA 23708 ENSG00000189369 GSPT2 G1 to S phase transition 2

7086 NA 7086 ENSG00000163931 TKT transketolase

5715 NA 5715 ENSG00000110801 PSMD9 proteasome (prosome, macropain) 26S subunit, non-ATPase, 9

140465 NA 140465 ENSG00000196465 MYL6B myosin, light chain 6B, alkali, smooth muscle and non-muscle

5478 NA 5478 ENSG00000196262 PPIA peptidylprolyl isomerase A (cyclophilin A)

4000 NA 4000 ENSG00000160789 LMNA lamin A/C

3692 NA 3692 ENSG00000126005 EIF6 eukaryotic translation initiation factor 6

3157 NA 3157 ENSG00000112972 HMGCS1 3-hydroxy-3-methylglutaryl-Coenzyme A synthase 1 (soluble)

1938 NA 1938 ENSG00000167658 EEF2 eukaryotic translation elongation factor 2

6432 NA 6432 ENSG00000115875 SFRS7 splicing factor, arginine/serine-rich 7, 35kDa

11164 NA 11164 ENSG00000165609 NUDT5 nudix (nucleoside diphosphate linked moiety X)-type motif 5

3320 NA 3320 ENSG00000080824 HSP90AA1 heat shock protein 90kDa alpha (cytosolic), class A member 1

10728 NA 10728 ENSG00000110958 PTGES3 prostaglandin E synthase 3 (cytosolic)

8407 NA 8407 ENSG00000158710 TAGLN2 transgelin 2

4634 NA 4634 ENSG00000160808 MYL3 myosin, light chain 3, alkali; ventricular, skeletal, slow

1984 NA 1984 ENSG00000132507 EIF5A eukaryotic translation initiation factor 5A

1975 NA 1975 ENSG00000063046 EIF4B eukaryotic translation initiation factor 4B

1892 NA 1892 ENSG00000127884 ECHS1 enoyl Coenzyme A hydratase, short chain, 1, mitochondrial

5315 NA 5315 ENSG00000067225 PKM2 pyruvate kinase, muscle

7520 NA 7520 ENSG00000079246 XRCC5 X-ray repair complementing defective repair in Chinese hamster cells 5 (double-strand-break rejoining)

11335 NA 11335 ENSG00000122565 CBX3 chromobox homolog 3 (HP1 gamma homolog, Drosophila)

3313 NA 3313 ENSG00000113013 HSPA9 heat shock 70kDa protein 9 (mortalin)

6624 NA 6624 ENSG00000075618 FSCN1 fascin homolog 1, actin-bundling protein (Strongylocentrotus purpuratus)

8570 NA 8570 ENSG00000088247 KHSRP KH-type splicing regulatory protein

2745 NA 2745 ENSG00000173221 GLRX glutaredoxin (thioltransferase)

6428 NA 6428 ENSG00000112081 SFRS3 splicing factor, arginine/serine-rich 3

6206 NA 6206 ENSG00000112306 RPS12 ribosomal protein S12

832 NA 832 ENSG00000077549 CAPZB capping protein (actin filament) muscle Z-line, beta

8683 NA 8683 ENSG00000111786 SFRS9 splicing factor, arginine/serine-rich 9

cellular component intracellular part GO:0044424

C=10572;O=67;E=44.58;R=1.50;rawP=1.16e-11;adjP=6.15e-10

7295 NA 7295 ENSG00000136810 TXN thioredoxin

3068 NA 3068 ENSG00000143321 HDGF hepatoma-derived growth factor (high-mobility group protein 1-like)

1674 NA 1674 ENSG00000175084 DES desmin

10383 NA 10383 ENSG00000188229 TUBB2C tubulin, beta 2C

4869 NA 4869 ENSG00000181163 NPM1 nucleophosmin (nucleolar phosphoprotein B23, numatrin)

5093 NA 5093 ENSG00000169564 PCBP1 poly(rC) binding protein 1

1937 NA 1937 ENSG00000149016 EEF1G eukaryotic translation elongation factor 1 gamma

220988 NA 220988 ENSG00000170144 HNRNPA3 heterogeneous nuclear ribonucleoprotein A3

11315 NA 11315 ENSG00000116288 PARK7 Parkinson disease (autosomal recessive, early onset) 7

3868 NA 3868 ENSG00000186832 KRT16 keratin 16

1933 NA 1933 ENSG00000114942 EEF1B2 eukaryotic translation elongation factor 1 beta 2

79174 NA 79174 ENSG00000184164 CRELD2 cysteine-rich with EGF-like domains 2

1854 NA 1854 ENSG00000128951 DUT deoxyuridine triphosphatase

4831 NA 4831 ENSG00000011052 NME2 non-metastatic cells 2, protein (NM23B) expressed in

6434 NA 6434 ENSG00000136527 TRA2B transformer 2 beta homolog (Drosophila)

4637 NA 4637 ENSG00000092841 MYL6 myosin, light chain 6, alkali, smooth muscle and non-muscle

3191 NA 3191 ENSG00000104824 HNRNPL heterogeneous nuclear ribonucleoprotein L

136319 NA 136319 ENSG00000105887 MTPN myotrophin

1072 NA 1072 ENSG00000172757 CFL1 cofilin 1 (non-muscle)

5094 NA 5094 ENSG00000197111 PCBP2 poly(rC) binding protein 2

2617 NA 2617 ENSG00000106105 GARS glycyl-tRNA synthetase

4841 NA 4841 ENSG00000147140 NONO non-POU domain containing, octamer-binding

6275 NA 6275 ENSG00000196154 S100A4 S100 calcium binding protein A4

3326 NA 3326 ENSG00000096384 HSP90AB1 heat shock protein 90kDa alpha (cytosolic), class B member 1

475 NA 475 ENSG00000177556 ATOX1 ATX1 antioxidant protein 1 homolog (yeast)

7417 NA 7417 ENSG00000165637 VDAC2 voltage-dependent anion channel 2

3956 NA 3956 ENSG00000100097 LGALS1 lectin, galactoside-binding, soluble, 1

10971 NA 10971 ENSG00000134308 YWHAQ tyrosine 3-monooxygenase/tryptophan 5-monooxygenase activation protein, theta polypeptide

7431 NA 7431 ENSG00000026025 VIM vimentin

1207 NA 1207 ENSG00000074201 CLNS1A chloride channel, nucleotide-sensitive, 1A

6426 NA 6426 ENSG00000136450 SFRS1 splicing factor, arginine/serine-rich 1

23589 NA 23589 ENSG00000153048 CARHSP1 calcium regulated heat stable protein 1, 24kDa

4830 NA 4830 ENSG00000011052 NME1 non-metastatic cells 1, protein (NM23A) expressed in

55250 NA 55250 ENSG00000134759 ELP2 elongation protein 2 homolog (S. cerevisiae)

6176 NA 6176 ENSG00000137818 RPLP1 ribosomal protein, large, P1

52 NA 52 ENSG00000143727 ACP1 acid phosphatase 1, soluble

3315 NA 3315 ENSG00000106211 HSPB1 heat shock 27kDa protein 1

203068 NA 203068 ENSG00000196230 TUBB tubulin, beta

3146 NA 3146 ENSG00000189403 HMGB1 high-mobility group box 1

23708 NA 23708 ENSG00000189369 GSPT2 G1 to S phase transition 2

7086 NA 7086 ENSG00000163931 TKT transketolase

5715 NA 5715 ENSG00000110801 PSMD9 proteasome (prosome, macropain) 26S subunit, non-ATPase, 9

140465 NA 140465 ENSG00000196465 MYL6B myosin, light chain 6B, alkali, smooth muscle and non-muscle

5478 NA 5478 ENSG00000196262 PPIA peptidylprolyl isomerase A (cyclophilin A)

4000 NA 4000 ENSG00000160789 LMNA lamin A/C

3692 NA 3692 ENSG00000126005 EIF6 eukaryotic translation initiation factor 6

3157 NA 3157 ENSG00000112972 HMGCS1 3-hydroxy-3-methylglutaryl-Coenzyme A synthase 1 (soluble)

1938 NA 1938 ENSG00000167658 EEF2 eukaryotic translation elongation factor 2

6432 NA 6432 ENSG00000115875 SFRS7 splicing factor, arginine/serine-rich 7, 35kDa

3320 NA 3320 ENSG00000080824 HSP90AA1 heat shock protein 90kDa alpha (cytosolic), class A member 1

10728 NA 10728 ENSG00000110958 PTGES3 prostaglandin E synthase 3 (cytosolic)

8407 NA 8407 ENSG00000158710 TAGLN2 transgelin 2

4634 NA 4634 ENSG00000160808 MYL3 myosin, light chain 3, alkali; ventricular, skeletal, slow

1984 NA 1984 ENSG00000132507 EIF5A eukaryotic translation initiation factor 5A

1975 NA 1975 ENSG00000063046 EIF4B eukaryotic translation initiation factor 4B

1892 NA 1892 ENSG00000127884 ECHS1 enoyl Coenzyme A hydratase, short chain, 1, mitochondrial

5315 NA 5315 ENSG00000067225 PKM2 pyruvate kinase, muscle

7520 NA 7520 ENSG00000079246 XRCC5 X-ray repair complementing defective repair in Chinese hamster cells 5 (double-strand-break rejoining)

11335 NA 11335 ENSG00000122565 CBX3 chromobox homolog 3 (HP1 gamma homolog, Drosophila)

3313 NA 3313 ENSG00000113013 HSPA9 heat shock 70kDa protein 9 (mortalin)

6624 NA 6624 ENSG00000075618 FSCN1 fascin homolog 1, actin-bundling protein (Strongylocentrotus purpuratus)

8570 NA 8570 ENSG00000088247 KHSRP KH-type splicing regulatory protein

2745 NA 2745 ENSG00000173221 GLRX glutaredoxin (thioltransferase)

6428 NA 6428 ENSG00000112081 SFRS3 splicing factor, arginine/serine-rich 3

6206 NA 6206 ENSG00000112306 RPS12 ribosomal protein S12

832 NA 832 ENSG00000077549 CAPZB capping protein (actin filament) muscle Z-line, beta

8683 NA 8683 ENSG00000111786 SFRS9 splicing factor, arginine/serine-rich 9

cellular component cytosol GO:0005829

C=1251;O=24;E=5.27;R=4.55;rawP=9.82e-11;adjP=3.47e-09

1984 NA 1984 ENSG00000132507 EIF5A eukaryotic translation initiation factor 5A

2617 NA 2617 ENSG00000106105 GARS glycyl-tRNA synthetase

7295 NA 7295 ENSG00000136810 TXN thioredoxin

1975 NA 1975 ENSG00000063046 EIF4B eukaryotic translation initiation factor 4B

10383 NA 10383 ENSG00000188229 TUBB2C tubulin, beta 2C

475 NA 475 ENSG00000177556 ATOX1 ATX1 antioxidant protein 1 homolog (yeast)

7086 NA 7086 ENSG00000163931 TKT transketolase

4869 NA 4869 ENSG00000181163 NPM1 nucleophosmin (nucleolar phosphoprotein B23, numatrin)

7431 NA 7431 ENSG00000026025 VIM vimentin

5315 NA 5315 ENSG00000067225 PKM2 pyruvate kinase, muscle

1937 NA 1937 ENSG00000149016 EEF1G eukaryotic translation elongation factor 1 gamma

11315 NA 11315 ENSG00000116288 PARK7 Parkinson disease (autosomal recessive, early onset) 7

5478 NA 5478 ENSG00000196262 PPIA peptidylprolyl isomerase A (cyclophilin A)

1207 NA 1207 ENSG00000074201 CLNS1A chloride channel, nucleotide-sensitive, 1A

1933 NA 1933 ENSG00000114942 EEF1B2 eukaryotic translation elongation factor 1 beta 2

3157 NA 3157 ENSG00000112972 HMGCS1 3-hydroxy-3-methylglutaryl-Coenzyme A synthase 1 (soluble)

4830 NA 4830 ENSG00000011052 NME1 non-metastatic cells 1, protein (NM23A) expressed in

1854 NA 1854 ENSG00000128951 DUT deoxyuridine triphosphatase

6176 NA 6176 ENSG00000137818 RPLP1 ribosomal protein, large, P1

2745 NA 2745 ENSG00000173221 GLRX glutaredoxin (thioltransferase)

6206 NA 6206 ENSG00000112306 RPS12 ribosomal protein S12

10728 NA 10728 ENSG00000110958 PTGES3 prostaglandin E synthase 3 (cytosolic)

3320 NA 3320 ENSG00000080824 HSP90AA1 heat shock protein 90kDa alpha (cytosolic), class A member 1

203068 NA 203068 ENSG00000196230 TUBB tubulin, beta

cellular component cytoplasm GO:0005737

C=7246;O=55;E=30.55;R=1.80;rawP=1.19e-09;adjP=3.15e-08

7295 NA 7295 ENSG00000136810 TXN thioredoxin

3068 NA 3068 ENSG00000143321 HDGF hepatoma-derived growth factor (high-mobility group protein 1-like)

1674 NA 1674 ENSG00000175084 DES desmin

10383 NA 10383 ENSG00000188229 TUBB2C tubulin, beta 2C

23708 NA 23708 ENSG00000189369 GSPT2 G1 to S phase transition 2

7086 NA 7086 ENSG00000163931 TKT transketolase

4869 NA 4869 ENSG00000181163 NPM1 nucleophosmin (nucleolar phosphoprotein B23, numatrin)

5093 NA 5093 ENSG00000169564 PCBP1 poly(rC) binding protein 1

1937 NA 1937 ENSG00000149016 EEF1G eukaryotic translation elongation factor 1 gamma

140465 NA 140465 ENSG00000196465 MYL6B myosin, light chain 6B, alkali, smooth muscle and non-muscle

220988 NA 220988 ENSG00000170144 HNRNPA3 heterogeneous nuclear ribonucleoprotein A3

11315 NA 11315 ENSG00000116288 PARK7 Parkinson disease (autosomal recessive, early onset) 7

4000 NA 4000 ENSG00000160789 LMNA lamin A/C

5478 NA 5478 ENSG00000196262 PPIA peptidylprolyl isomerase A (cyclophilin A)

1933 NA 1933 ENSG00000114942 EEF1B2 eukaryotic translation elongation factor 1 beta 2

3692 NA 3692 ENSG00000126005 EIF6 eukaryotic translation initiation factor 6

79174 NA 79174 ENSG00000184164 CRELD2 cysteine-rich with EGF-like domains 2

3157 NA 3157 ENSG00000112972 HMGCS1 3-hydroxy-3-methylglutaryl-Coenzyme A synthase 1 (soluble)

1938 NA 1938 ENSG00000167658 EEF2 eukaryotic translation elongation factor 2

1854 NA 1854 ENSG00000128951 DUT deoxyuridine triphosphatase

4831 NA 4831 ENSG00000011052 NME2 non-metastatic cells 2, protein (NM23B) expressed in

10728 NA 10728 ENSG00000110958 PTGES3 prostaglandin E synthase 3 (cytosolic)

3320 NA 3320 ENSG00000080824 HSP90AA1 heat shock protein 90kDa alpha (cytosolic), class A member 1

136319 NA 136319 ENSG00000105887 MTPN myotrophin

5094 NA 5094 ENSG00000197111 PCBP2 poly(rC) binding protein 2

1072 NA 1072 ENSG00000172757 CFL1 cofilin 1 (non-muscle)

4634 NA 4634 ENSG00000160808 MYL3 myosin, light chain 3, alkali; ventricular, skeletal, slow

1984 NA 1984 ENSG00000132507 EIF5A eukaryotic translation initiation factor 5A

2617 NA 2617 ENSG00000106105 GARS glycyl-tRNA synthetase

1975 NA 1975 ENSG00000063046 EIF4B eukaryotic translation initiation factor 4B

6275 NA 6275 ENSG00000196154 S100A4 S100 calcium binding protein A4

3326 NA 3326 ENSG00000096384 HSP90AB1 heat shock protein 90kDa alpha (cytosolic), class B member 1

475 NA 475 ENSG00000177556 ATOX1 ATX1 antioxidant protein 1 homolog (yeast)

1892 NA 1892 ENSG00000127884 ECHS1 enoyl Coenzyme A hydratase, short chain, 1, mitochondrial

7417 NA 7417 ENSG00000165637 VDAC2 voltage-dependent anion channel 2

3956 NA 3956 ENSG00000100097 LGALS1 lectin, galactoside-binding, soluble, 1

10971 NA 10971 ENSG00000134308 YWHAQ tyrosine 3-monooxygenase/tryptophan 5-monooxygenase activation protein, theta polypeptide

7431 NA 7431 ENSG00000026025 VIM vimentin

5315 NA 5315 ENSG00000067225 PKM2 pyruvate kinase, muscle

1207 NA 1207 ENSG00000074201 CLNS1A chloride channel, nucleotide-sensitive, 1A

7520 NA 7520 ENSG00000079246 XRCC5 X-ray repair complementing defective repair in Chinese hamster cells 5 (double-strand-break rejoining)

6426 NA 6426 ENSG00000136450 SFRS1 splicing factor, arginine/serine-rich 1

23589 NA 23589 ENSG00000153048 CARHSP1 calcium regulated heat stable protein 1, 24kDa

3313 NA 3313 ENSG00000113013 HSPA9 heat shock 70kDa protein 9 (mortalin)

55250 NA 55250 ENSG00000134759 ELP2 elongation protein 2 homolog (S. cerevisiae)

4830 NA 4830 ENSG00000011052 NME1 non-metastatic cells 1, protein (NM23A) expressed in

6624 NA 6624 ENSG00000075618 FSCN1 fascin homolog 1, actin-bundling protein (Strongylocentrotus purpuratus)

6176 NA 6176 ENSG00000137818 RPLP1 ribosomal protein, large, P1

8570 NA 8570 ENSG00000088247 KHSRP KH-type splicing regulatory protein

52 NA 52 ENSG00000143727 ACP1 acid phosphatase 1, soluble

2745 NA 2745 ENSG00000173221 GLRX glutaredoxin (thioltransferase)

6206 NA 6206 ENSG00000112306 RPS12 ribosomal protein S12

832 NA 832 ENSG00000077549 CAPZB capping protein (actin filament) muscle Z-line, beta

3315 NA 3315 ENSG00000106211 HSPB1 heat shock 27kDa protein 1

203068 NA 203068 ENSG00000196230 TUBB tubulin, beta

cellular component macromolecular complex GO:0032991

C=3040;O=32;E=12.82;R=2.50;rawP=1.10e-07;adjP=2.33e-06

1984 NA 1984 ENSG00000132507 EIF5A eukaryotic translation initiation factor 5A

1975 NA 1975 ENSG00000063046 EIF4B eukaryotic translation initiation factor 4B

1674 NA 1674 ENSG00000175084 DES desmin

10383 NA 10383 ENSG00000188229 TUBB2C tubulin, beta 2C

7417 NA 7417 ENSG00000165637 VDAC2 voltage-dependent anion channel 2

10971 NA 10971 ENSG00000134308 YWHAQ tyrosine 3-monooxygenase/tryptophan 5-monooxygenase activation protein, theta polypeptide

4869 NA 4869 ENSG00000181163 NPM1 nucleophosmin (nucleolar phosphoprotein B23, numatrin)

7431 NA 7431 ENSG00000026025 VIM vimentin

5715 NA 5715 ENSG00000110801 PSMD9 proteasome (prosome, macropain) 26S subunit, non-ATPase, 9

5093 NA 5093 ENSG00000169564 PCBP1 poly(rC) binding protein 1

1937 NA 1937 ENSG00000149016 EEF1G eukaryotic translation elongation factor 1 gamma

140465 NA 140465 ENSG00000196465 MYL6B myosin, light chain 6B, alkali, smooth muscle and non-muscle

220988 NA 220988 ENSG00000170144 HNRNPA3 heterogeneous nuclear ribonucleoprotein A3

4000 NA 4000 ENSG00000160789 LMNA lamin A/C

3868 NA 3868 ENSG00000186832 KRT16 keratin 16

7520 NA 7520 ENSG00000079246 XRCC5 X-ray repair complementing defective repair in Chinese hamster cells 5 (double-strand-break rejoining)

6426 NA 6426 ENSG00000136450 SFRS1 splicing factor, arginine/serine-rich 1

1933 NA 1933 ENSG00000114942 EEF1B2 eukaryotic translation elongation factor 1 beta 2

3692 NA 3692 ENSG00000126005 EIF6 eukaryotic translation initiation factor 6

1938 NA 1938 ENSG00000167658 EEF2 eukaryotic translation elongation factor 2

55250 NA 55250 ENSG00000134759 ELP2 elongation protein 2 homolog (S. cerevisiae)

4830 NA 4830 ENSG00000011052 NME1 non-metastatic cells 1, protein (NM23A) expressed in

6176 NA 6176 ENSG00000137818 RPLP1 ribosomal protein, large, P1

4637 NA 4637 ENSG00000092841 MYL6 myosin, light chain 6, alkali, smooth muscle and non-muscle

3191 NA 3191 ENSG00000104824 HNRNPL heterogeneous nuclear ribonucleoprotein L

6206 NA 6206 ENSG00000112306 RPS12 ribosomal protein S12

10728 NA 10728 ENSG00000110958 PTGES3 prostaglandin E synthase 3 (cytosolic)

832 NA 832 ENSG00000077549 CAPZB capping protein (actin filament) muscle Z-line, beta

3315 NA 3315 ENSG00000106211 HSPB1 heat shock 27kDa protein 1

203068 NA 203068 ENSG00000196230 TUBB tubulin, beta

5094 NA 5094 ENSG00000197111 PCBP2 poly(rC) binding protein 2

4634 NA 4634 ENSG00000160808 MYL3 myosin, light chain 3, alkali; ventricular, skeletal, slow

cellular component non-membrane-bounded organelle GO:0043228

C=2547;O=28;E=10.74;R=2.61;rawP=4.65e-07;adjP=7.04e-06

3146 NA 3146 ENSG00000189403 HMGB1 high-mobility group box 1

1674 NA 1674 ENSG00000175084 DES desmin

10383 NA 10383 ENSG00000188229 TUBB2C tubulin, beta 2C

6275 NA 6275 ENSG00000196154 S100A4 S100 calcium binding protein A4

7417 NA 7417 ENSG00000165637 VDAC2 voltage-dependent anion channel 2

4869 NA 4869 ENSG00000181163 NPM1 nucleophosmin (nucleolar phosphoprotein B23, numatrin)

7431 NA 7431 ENSG00000026025 VIM vimentin

140465 NA 140465 ENSG00000196465 MYL6B myosin, light chain 6B, alkali, smooth muscle and non-muscle

220988 NA 220988 ENSG00000170144 HNRNPA3 heterogeneous nuclear ribonucleoprotein A3

4000 NA 4000 ENSG00000160789 LMNA lamin A/C

1207 NA 1207 ENSG00000074201 CLNS1A chloride channel, nucleotide-sensitive, 1A

3868 NA 3868 ENSG00000186832 KRT16 keratin 16

11335 NA 11335 ENSG00000122565 CBX3 chromobox homolog 3 (HP1 gamma homolog, Drosophila)

7520 NA 7520 ENSG00000079246 XRCC5 X-ray repair complementing defective repair in Chinese hamster cells 5 (double-strand-break rejoining)

3692 NA 3692 ENSG00000126005 EIF6 eukaryotic translation initiation factor 6

3313 NA 3313 ENSG00000113013 HSPA9 heat shock 70kDa protein 9 (mortalin)

55250 NA 55250 ENSG00000134759 ELP2 elongation protein 2 homolog (S. cerevisiae)

4830 NA 4830 ENSG00000011052 NME1 non-metastatic cells 1, protein (NM23A) expressed in

6624 NA 6624 ENSG00000075618 FSCN1 fascin homolog 1, actin-bundling protein (Strongylocentrotus purpuratus)

6176 NA 6176 ENSG00000137818 RPLP1 ribosomal protein, large, P1

4637 NA 4637 ENSG00000092841 MYL6 myosin, light chain 6, alkali, smooth muscle and non-muscle

6206 NA 6206 ENSG00000112306 RPS12 ribosomal protein S12

10728 NA 10728 ENSG00000110958 PTGES3 prostaglandin E synthase 3 (cytosolic)

832 NA 832 ENSG00000077549 CAPZB capping protein (actin filament) muscle Z-line, beta

3315 NA 3315 ENSG00000106211 HSPB1 heat shock 27kDa protein 1

1072 NA 1072 ENSG00000172757 CFL1 cofilin 1 (non-muscle)

203068 NA 203068 ENSG00000196230 TUBB tubulin, beta

4634 NA 4634 ENSG00000160808 MYL3 myosin, light chain 3, alkali; ventricular, skeletal, slow

cellular component intracellular non-membrane-bounded organelle GO:0043232

C=2547;O=28;E=10.74;R=2.61;rawP=4.65e-07;adjP=7.04e-06

3146 NA 3146 ENSG00000189403 HMGB1 high-mobility group box 1

1674 NA 1674 ENSG00000175084 DES desmin

10383 NA 10383 ENSG00000188229 TUBB2C tubulin, beta 2C

6275 NA 6275 ENSG00000196154 S100A4 S100 calcium binding protein A4

7417 NA 7417 ENSG00000165637 VDAC2 voltage-dependent anion channel 2

4869 NA 4869 ENSG00000181163 NPM1 nucleophosmin (nucleolar phosphoprotein B23, numatrin)

7431 NA 7431 ENSG00000026025 VIM vimentin

140465 NA 140465 ENSG00000196465 MYL6B myosin, light chain 6B, alkali, smooth muscle and non-muscle

220988 NA 220988 ENSG00000170144 HNRNPA3 heterogeneous nuclear ribonucleoprotein A3

4000 NA 4000 ENSG00000160789 LMNA lamin A/C

1207 NA 1207 ENSG00000074201 CLNS1A chloride channel, nucleotide-sensitive, 1A

3868 NA 3868 ENSG00000186832 KRT16 keratin 16

11335 NA 11335 ENSG00000122565 CBX3 chromobox homolog 3 (HP1 gamma homolog, Drosophila)

7520 NA 7520 ENSG00000079246 XRCC5 X-ray repair complementing defective repair in Chinese hamster cells 5 (double-strand-break rejoining)

3692 NA 3692 ENSG00000126005 EIF6 eukaryotic translation initiation factor 6

3313 NA 3313 ENSG00000113013 HSPA9 heat shock 70kDa protein 9 (mortalin)

55250 NA 55250 ENSG00000134759 ELP2 elongation protein 2 homolog (S. cerevisiae)

4830 NA 4830 ENSG00000011052 NME1 non-metastatic cells 1, protein (NM23A) expressed in

6624 NA 6624 ENSG00000075618 FSCN1 fascin homolog 1, actin-bundling protein (Strongylocentrotus purpuratus)

6176 NA 6176 ENSG00000137818 RPLP1 ribosomal protein, large, P1

4637 NA 4637 ENSG00000092841 MYL6 myosin, light chain 6, alkali, smooth muscle and non-muscle

6206 NA 6206 ENSG00000112306 RPS12 ribosomal protein S12

10728 NA 10728 ENSG00000110958 PTGES3 prostaglandin E synthase 3 (cytosolic)

832 NA 832 ENSG00000077549 CAPZB capping protein (actin filament) muscle Z-line, beta

3315 NA 3315 ENSG00000106211 HSPB1 heat shock 27kDa protein 1

1072 NA 1072 ENSG00000172757 CFL1 cofilin 1 (non-muscle)

203068 NA 203068 ENSG00000196230 TUBB tubulin, beta

4634 NA 4634 ENSG00000160808 MYL3 myosin, light chain 3, alkali; ventricular, skeletal, slow

cellular component cytoskeletal part GO:0044430

C=927;O=16;E=3.91;R=4.09;rawP=1.09e-06;adjP=1.44e-05

3692 NA 3692 ENSG00000126005 EIF6 eukaryotic translation initiation factor 6

4830 NA 4830 ENSG00000011052 NME1 non-metastatic cells 1, protein (NM23A) expressed in

1674 NA 1674 ENSG00000175084 DES desmin

10383 NA 10383 ENSG00000188229 TUBB2C tubulin, beta 2C

4869 NA 4869 ENSG00000181163 NPM1 nucleophosmin (nucleolar phosphoprotein B23, numatrin)

4637 NA 4637 ENSG00000092841 MYL6 myosin, light chain 6, alkali, smooth muscle and non-muscle

7431 NA 7431 ENSG00000026025 VIM vimentin

832 NA 832 ENSG00000077549 CAPZB capping protein (actin filament) muscle Z-line, beta

3315 NA 3315 ENSG00000106211 HSPB1 heat shock 27kDa protein 1

140465 NA 140465 ENSG00000196465 MYL6B myosin, light chain 6B, alkali, smooth muscle and non-muscle

4000 NA 4000 ENSG00000160789 LMNA lamin A/C

203068 NA 203068 ENSG00000196230 TUBB tubulin, beta

3868 NA 3868 ENSG00000186832 KRT16 keratin 16

1072 NA 1072 ENSG00000172757 CFL1 cofilin 1 (non-muscle)

11335 NA 11335 ENSG00000122565 CBX3 chromobox homolog 3 (HP1 gamma homolog, Drosophila)

4634 NA 4634 ENSG00000160808 MYL3 myosin, light chain 3, alkali; ventricular, skeletal, slow

cellular component cytoskeleton GO:0005856

C=1353;O=18;E=5.70;R=3.16;rawP=8.34e-06;adjP=9.82e-05

1674 NA 1674 ENSG00000175084 DES desmin

10383 NA 10383 ENSG00000188229 TUBB2C tubulin, beta 2C

4869 NA 4869 ENSG00000181163 NPM1 nucleophosmin (nucleolar phosphoprotein B23, numatrin)

7431 NA 7431 ENSG00000026025 VIM vimentin

140465 NA 140465 ENSG00000196465 MYL6B myosin, light chain 6B, alkali, smooth muscle and non-muscle

4000 NA 4000 ENSG00000160789 LMNA lamin A/C

3868 NA 3868 ENSG00000186832 KRT16 keratin 16

1207 NA 1207 ENSG00000074201 CLNS1A chloride channel, nucleotide-sensitive, 1A

11335 NA 11335 ENSG00000122565 CBX3 chromobox homolog 3 (HP1 gamma homolog, Drosophila)

3692 NA 3692 ENSG00000126005 EIF6 eukaryotic translation initiation factor 6

4830 NA 4830 ENSG00000011052 NME1 non-metastatic cells 1, protein (NM23A) expressed in

6624 NA 6624 ENSG00000075618 FSCN1 fascin homolog 1, actin-bundling protein (Strongylocentrotus purpuratus)

4637 NA 4637 ENSG00000092841 MYL6 myosin, light chain 6, alkali, smooth muscle and non-muscle

832 NA 832 ENSG00000077549 CAPZB capping protein (actin filament) muscle Z-line, beta

3315 NA 3315 ENSG00000106211 HSPB1 heat shock 27kDa protein 1

1072 NA 1072 ENSG00000172757 CFL1 cofilin 1 (non-muscle)

203068 NA 203068 ENSG00000196230 TUBB tubulin, beta

4634 NA 4634 ENSG00000160808 MYL3 myosin, light chain 3, alkali; ventricular, skeletal, slow

cellular component ribonucleoprotein complex GO:0030529

C=496;O=10;E=2.09;R=4.78;rawP=4.06e-05;adjP=0.0004

1938 NA 1938 ENSG00000167658 EEF2 eukaryotic translation elongation factor 2

6176 NA 6176 ENSG00000137818 RPLP1 ribosomal protein, large, P1

4869 NA 4869 ENSG00000181163 NPM1 nucleophosmin (nucleolar phosphoprotein B23, numatrin)

3191 NA 3191 ENSG00000104824 HNRNPL heterogeneous nuclear ribonucleoprotein L

10728 NA 10728 ENSG00000110958 PTGES3 prostaglandin E synthase 3 (cytosolic)

6206 NA 6206 ENSG00000112306 RPS12 ribosomal protein S12

5093 NA 5093 ENSG00000169564 PCBP1 poly(rC) binding protein 1

220988 NA 220988 ENSG00000170144 HNRNPA3 heterogeneous nuclear ribonucleoprotein A3

5094 NA 5094 ENSG00000197111 PCBP2 poly(rC) binding protein 2

6426 NA 6426 ENSG00000136450 SFRS1 splicing factor, arginine/serine-rich 1

# KEGG Pathway Analysis

User file and parameters: User file: PROP_h2o2_entrezgene.txt, Organism: hsapiens, Id Type: entrezgene, Ref Set: entrezgene, Significance Level: Top10, Statistics Test: Hypergeometric, MTC: BH, Minimum: 2

The results for each enriched KEGG pathway are listed in this table. For each pathway, the first row lists its KEGG pathway

name, and corresponding KEGG ID. The second row lists

number of reference genes in the category (C), number of genes in the gene set and also in the category (O), expected number in the

category (E), Ratio of enrichment (R), p value from hypergeometric test (rawP), and p value adjusted by the multiple test adjustment (adjP).

Finally, genes in the pathway are listed. For each gene, the table lists the user uploaded ID and value (optional), Entrez ID,

Ensembl Gene Stable ID, Gene symbol, and description.

KEGG pathway Spliceosome 03040

C=128;O=7;E=0.20;R=35.42;rawP=1.25e-09;adjP=2.38e-08

6434 NA 6434 ENSG00000136527 TRA2B transformer 2 beta homolog (Drosophila)

6428 NA 6428 ENSG00000112081 SFRS3 splicing factor, arginine/serine-rich 3

5093 NA 5093 ENSG00000169564 PCBP1 poly(rC) binding protein 1

220988 NA 220988 ENSG00000170144 HNRNPA3 heterogeneous nuclear ribonucleoprotein A3

8683 NA 8683 ENSG00000111786 SFRS9 splicing factor, arginine/serine-rich 9

6426 NA 6426 ENSG00000136450 SFRS1 splicing factor, arginine/serine-rich 1

6432 NA 6432 ENSG00000115875 SFRS7 splicing factor, arginine/serine-rich 7, 35kDa

KEGG pathway Purine metabolism 00230

C=151;O=5;E=0.23;R=21.45;rawP=3.90e-06;adjP=3.70e-05

11164 NA 11164 ENSG00000165609 NUDT5 nudix (nucleoside diphosphate linked moiety X)-type motif 5

4830 NA 4830 ENSG00000011052 NME1 non-metastatic cells 1, protein (NM23A) expressed in

5315 NA 5315 ENSG00000067225 PKM2 pyruvate kinase, muscle

10606 NA 10606 ENSG00000128050 PAICS phosphoribosylaminoimidazole carboxylase, phosphoribosylaminoimidazole succinocarboxamide synthetase

4831 NA 4831 ENSG00000011052 NME2 non-metastatic cells 2, protein (NM23B) expressed in

KEGG pathway Metabolic pathways 01100

C=1104;O=9;E=1.70;R=5.28;rawP=5.00e-05;adjP=0.0003

3157 NA 3157 ENSG00000112972 HMGCS1 3-hydroxy-3-methylglutaryl-Coenzyme A synthase 1 (soluble)

4830 NA 4830 ENSG00000011052 NME1 non-metastatic cells 1, protein (NM23A) expressed in

1854 NA 1854 ENSG00000128951 DUT deoxyuridine triphosphatase

10606 NA 10606 ENSG00000128050 PAICS phosphoribosylaminoimidazole carboxylase, phosphoribosylaminoimidazole succinocarboxamide synthetase

1892 NA 1892 ENSG00000127884 ECHS1 enoyl Coenzyme A hydratase, short chain, 1, mitochondrial

4831 NA 4831 ENSG00000011052 NME2 non-metastatic cells 2, protein (NM23B) expressed in

7086 NA 7086 ENSG00000163931 TKT transketolase

5315 NA 5315 ENSG00000067225 PKM2 pyruvate kinase, muscle

26227 NA 26227 ENSG00000092621 PHGDH phosphoglycerate dehydrogenase

KEGG pathway Pathogenic Escherichia coli infection 05130

C=59;O=3;E=0.09;R=32.93;rawP=0.0001;adjP=0.0005

10971 NA 10971 ENSG00000134308 YWHAQ tyrosine 3-monooxygenase/tryptophan 5-monooxygenase activation protein, theta polypeptide

10383 NA 10383 ENSG00000188229 TUBB2C tubulin, beta 2C

203068 NA 203068 ENSG00000196230 TUBB tubulin, beta

KEGG pathway Hypertrophic cardiomyopathy (HCM) 05410

C=85;O=3;E=0.13;R=22.86;rawP=0.0003;adjP=0.0011

1674 NA 1674 ENSG00000175084 DES desmin

4000 NA 4000 ENSG00000160789 LMNA lamin A/C

4634 NA 4634 ENSG00000160808 MYL3 myosin, light chain 3, alkali; ventricular, skeletal, slow

KEGG pathway Dilated cardiomyopathy 05414

C=92;O=3;E=0.14;R=21.12;rawP=0.0004;adjP=0.0013

1674 NA 1674 ENSG00000175084 DES desmin

4000 NA 4000 ENSG00000160789 LMNA lamin A/C

4634 NA 4634 ENSG00000160808 MYL3 myosin, light chain 3, alkali; ventricular, skeletal, slow

KEGG pathway Pyrimidine metabolism 00240

C=98;O=3;E=0.15;R=19.83;rawP=0.0005;adjP=0.0014

4830 NA 4830 ENSG00000011052 NME1 non-metastatic cells 1, protein (NM23A) expressed in

1854 NA 1854 ENSG00000128951 DUT deoxyuridine triphosphatase

4831 NA 4831 ENSG00000011052 NME2 non-metastatic cells 2, protein (NM23B) expressed in

KEGG pathway Butanoate metabolism 00650

C=34;O=2;E=0.05;R=38.10;rawP=0.0013;adjP=0.0031

3157 NA 3157 ENSG00000112972 HMGCS1 3-hydroxy-3-methylglutaryl-Coenzyme A synthase 1 (soluble)

1892 NA 1892 ENSG00000127884 ECHS1 enoyl Coenzyme A hydratase, short chain, 1, mitochondrial

KEGG pathway Valine, leucine and isoleucine degradation 00280

C=44;O=2;E=0.07;R=29.44;rawP=0.0021;adjP=0.0044

3157 NA 3157 ENSG00000112972 HMGCS1 3-hydroxy-3-methylglutaryl-Coenzyme A synthase 1 (soluble)

1892 NA 1892 ENSG00000127884 ECHS1 enoyl Coenzyme A hydratase, short chain, 1, mitochondrial

KEGG pathway NOD-like receptor signaling pathway 04621

C=62;O=2;E=0.10;R=20.89;rawP=0.0042;adjP=0.0080

3320 NA 3320 ENSG00000080824 HSP90AA1 heat shock protein 90kDa alpha (cytosolic), class A member 1

3326 NA 3326 ENSG00000096384 HSP90AB1 heat shock protein 90kDa alpha (cytosolic), class B member 1

# Wikipathways Analysis

User file and parameters: User file: PROP_h2o2_entrezgene.txt, Organism: hsapiens, Id Type: entrezgene, Ref Set: entrezgene, Significance Level: Top10, Statistics Test: Hypergeometric, MTC: BH, Minimum: 2

The results for each enriched gene sets are listed in this table. For each pathway, the first row lists its gene set

name, and corresponding Gene Set ID. The second row lists

number of reference genes in the category (C), number of genes in the gene set and also in the category (O), expected number in the

category (E), Ratio of enrichment (R), p value from hypergeometric test (rawP), and p value adjusted by the multiple test adjustment (adjP).

Finally, genes in the pathway are listed. For each gene, the table lists the user uploaded ID and value (optional), Entrez ID,

Ensembl Gene Stable ID, Gene symbol, and description.

Wikipathways pathway Translation Factors WP107

C=57;O=7;E=0.09;R=79.54;rawP=3.82e-12;adjP=4.58e-11

1984 NA 1984 ENSG00000132507 EIF5A eukaryotic translation initiation factor 5A

3692 NA 3692 ENSG00000126005 EIF6 eukaryotic translation initiation factor 6

1975 NA 1975 ENSG00000063046 EIF4B eukaryotic translation initiation factor 4B

1938 NA 1938 ENSG00000167658 EEF2 eukaryotic translation elongation factor 2

1937 NA 1937 ENSG00000149016 EEF1G eukaryotic translation elongation factor 1 gamma

23708 NA 23708 ENSG00000189369 GSPT2 G1 to S phase transition 2

1933 NA 1933 ENSG00000114942 EEF1B2 eukaryotic translation elongation factor 1 beta 2

Wikipathways pathway mRNA processing WP411

C=136;O=8;E=0.21;R=38.10;rawP=4.30e-11;adjP=2.58e-10

4841 NA 4841 ENSG00000147140 NONO non-POU domain containing, octamer-binding

6432 NA 6432 ENSG00000115875 SFRS7 splicing factor, arginine/serine-rich 7, 35kDa

6434 NA 6434 ENSG00000136527 TRA2B transformer 2 beta homolog (Drosophila)

3191 NA 3191 ENSG00000104824 HNRNPL heterogeneous nuclear ribonucleoprotein L

6428 NA 6428 ENSG00000112081 SFRS3 splicing factor, arginine/serine-rich 3

5094 NA 5094 ENSG00000197111 PCBP2 poly(rC) binding protein 2

8683 NA 8683 ENSG00000111786 SFRS9 splicing factor, arginine/serine-rich 9

6426 NA 6426 ENSG00000136450 SFRS1 splicing factor, arginine/serine-rich 1

Wikipathways pathway Striated Muscle Contraction WP383

C=37;O=3;E=0.06;R=52.52;rawP=2.64e-05;adjP=0.0001

7431 NA 7431 ENSG00000026025 VIM vimentin

1674 NA 1674 ENSG00000175084 DES desmin

4634 NA 4634 ENSG00000160808 MYL3 myosin, light chain 3, alkali; ventricular, skeletal, slow

Wikipathways pathway Alpha6-Beta4 Integrin Signaling Pathway WP244

C=71;O=3;E=0.11;R=27.37;rawP=0.0002;adjP=0.0005

10971 NA 10971 ENSG00000134308 YWHAQ tyrosine 3-monooxygenase/tryptophan 5-monooxygenase activation protein, theta polypeptide

7431 NA 7431 ENSG00000026025 VIM vimentin

3692 NA 3692 ENSG00000126005 EIF6 eukaryotic translation initiation factor 6

Wikipathways pathway TNF-alpha/NF-kB Signaling Pathway WP231

C=190;O=4;E=0.29;R=13.64;rawP=0.0002;adjP=0.0005

10971 NA 10971 ENSG00000134308 YWHAQ tyrosine 3-monooxygenase/tryptophan 5-monooxygenase activation protein, theta polypeptide

3320 NA 3320 ENSG00000080824 HSP90AA1 heat shock protein 90kDa alpha (cytosolic), class A member 1

3315 NA 3315 ENSG00000106211 HSPB1 heat shock 27kDa protein 1

3326 NA 3326 ENSG00000096384 HSP90AB1 heat shock protein 90kDa alpha (cytosolic), class B member 1

Wikipathways pathway Nucleotide Metabolism WP404

C=22;O=2;E=0.03;R=58.88;rawP=0.0005;adjP=0.0010

4830 NA 4830 ENSG00000011052 NME1 non-metastatic cells 1, protein (NM23A) expressed in

4831 NA 4831 ENSG00000011052 NME2 non-metastatic cells 2, protein (NM23B) expressed in

Wikipathways pathway T Cell Receptor Signaling Pathway WP69

C=134;O=3;E=0.21;R=14.50;rawP=0.0012;adjP=0.0021

52 NA 52 ENSG00000143727 ACP1 acid phosphatase 1, soluble

10971 NA 10971 ENSG00000134308 YWHAQ tyrosine 3-monooxygenase/tryptophan 5-monooxygenase activation protein, theta polypeptide

203068 NA 203068 ENSG00000196230 TUBB tubulin, beta

Wikipathways pathway FAS pathway and Stress induction of HSP regulation WP314

C=39;O=2;E=0.06;R=33.22;rawP=0.0017;adjP=0.0026

3315 NA 3315 ENSG00000106211 HSPB1 heat shock 27kDa protein 1

4000 NA 4000 ENSG00000160789 LMNA lamin A/C

Wikipathways pathway IL-6 Signaling Pathway WP364

C=98;O=2;E=0.15;R=13.22;rawP=0.0102;adjP=0.0127

3320 NA 3320 ENSG00000080824 HSP90AA1 heat shock protein 90kDa alpha (cytosolic), class A member 1

3315 NA 3315 ENSG00000106211 HSPB1 heat shock 27kDa protein 1

Wikipathways pathway IL-3 Signaling Pathway WP286

C=100;O=2;E=0.15;R=12.95;rawP=0.0106;adjP=0.0127

10971 NA 10971 ENSG00000134308 YWHAQ tyrosine 3-monooxygenase/tryptophan 5-monooxygenase activation protein, theta polypeptide

3315 NA 3315 ENSG00000106211 HSPB1 heat shock 27kDa protein 1
